# Simultaneous tracking of autophagy and oxidative stress during stroke with an ICT-TBET integrated ratiometric two-photon platform†

**DOI:** 10.1039/d1sc06805a

**Published:** 2022-04-21

**Authors:** Wei Hu, Taotao Qiang, Li Chai, Tianyu Liang, Longfang Ren, Fei Cheng, Chunya Li, Tony D. James

**Affiliations:** College of Bioresources and Materials Engineering, Shaanxi Collaborative Innovation Center of Industrial Auxiliary Chemistry & Technology, Shaanxi University of Science & Technology Xi'an 710021 China qiangtt515@163.com; Key Laboratory of Analytical Chemistry of the State Ethnic Affairs Commission, College of Chemistry and Material Science, South-Central University for Nationalities Wuhan 430074 China lichychem@mail.scuec.edu.cn; Department of Chemistry, University of Bath Bath BA27AY UK t.d.james@bath.ac.uk; School of Chemistry and Chemical Engineering, Henan Normal University Xinxiang 453007 China

## Abstract

Over recent years, fluorescent probes exhibiting simultaneous responses to multiple targets have been developed for *in situ*, real-time monitoring of cellular metabolism using two photon fluorescence sensing techniques due to numerous advantages including ease of operation, rapid reporting, high resolution, long visualization time and being non-invasive. However, due to interference from different fluorescence channels during simultaneous monitoring of multiple targets and the lack of ratiometric capability amongst the available probes, the accuracy in tracing metabolic processes has been restricted. With this research, using a through-bond energy transfer (TBET) mechanism, we designed a viscosity and peroxynitrite (ONOO^−^) mitochondria-targeting two-photon ratiometric fluorescent probe Mito-ONOO. Our results indicated that with decreasing levels of mitochondrial viscosity and increasing levels of ONOO^−^, the maximum of the emission wavelength of the probe shifted from 621 nm to 495 nm under 810 nm two-photon excitation. The baselines for the two emission peaks were significantly separated (Δ*λ* = 126 nm), improving the resolution and reliability of bioimaging. Moreover, by ratiometric analysis during oxygen-glucose deprivation/reoxygenation (OGD/R, commonly used to simulate cell ischemia/reperfusion injury), the real-time visualization of the metabolic processes of autophagy and oxidative stress was possible. Our research indicated that during cellular oxygen-glucose deprivation/reoxygenation, cells produce ONOO^−^, causing cellular oxidative stress and cellular autophagy after 15 min, as such Mito-ONOO exhibits the potential for the monitoring and diagnosis of stroke, as well as providing insight into potential treatments, and drug design.

## Abstract 2

Ratiometric simultaneous tracking of autophagy and oxidative stress was achieved using an ICT-TBET integrated platform. Mito-ONOO exhibited excellent selectivity, good chemical stability, and non-overlapping ratiometric signals.

## Graphical abstract

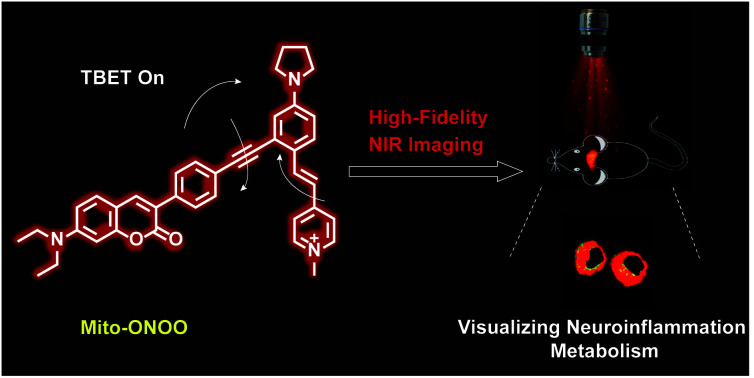

## Introduction

Cellular metabolism, including energy metabolism and substance metabolism, is a general term for chemical reactions within cells required to maintain life. Cellular metabolism provides metabolic intermediates for other biological components such as proteins and regulates intercellular signaling pathways.^1–3^ More importantly, the study of cellular metabolism enables the evaluation of common human diseases and enables the precise monitoring of relationships between cellular metabolism and disease states.^4–9^ As a key link between intrinsic regulation and phenotype of the organism, endogenous metabolites carry metabolic information. Therefore, *in situ* analysis with high temporal–spatial resolution and monitoring of multiple endogenous metabolites in a cellular metabolic process is vital in the areas of biology, pathology, and medicine.^10^ However, traditional biochemical analysis methods such as cell lysis, enzymology, chromatography, and mass spectrometry cannot monitor intracellular metabolism in real-time.

With the development of confocal imaging, *in situ*, real-time monitoring of cellular metabolism using fluorescent probes exhibiting simultaneous responses to multiple targets has resulted in significant advances.^11,12^ In particular, two-photon confocal technology using focused excitation can significantly improve the imaging resolution and visualization time.^13,14^ Single emission probes are affected by many factors, such as light source intensity, solvent scattering, color and viscosity, probe distribution, instrumental performance and so on, which results in inaccuracy, especially in the determination of the starting point of metabolism.^15^ As such there is a need to develop improved multi emission probes (ratiometric probes) with reduced crosstalk. Crosstalk can be reduced by large separation of the emission signals or by using multiple excitation wavelengths.^11^

**Scheme 1:**
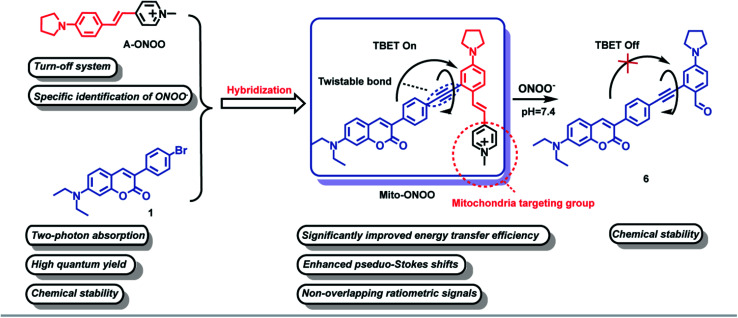
Structure of Mito-ONOO probe and its sensing mechanism towards ONOO^−^.

Through-bond energy transfer (TBET)-based fluorescent probes control energy transfer by regulating the energy level gap of the energy acceptors through chemical reactions, as such this mechanism has become a classic strategy for constructing ratiometric fluorescent probes. In addition, since the TBET mechanism does not require spectral overlap between energy donors and acceptors, and only requires a difference in the energy gap, baseline separation of the two emission signals can be achieved, thus reducing crosstalk between emissions. In addition, the high energy transfer efficiency of the TBET process can significantly enhance the sensitivity and accuracy of detection.^16^ All the above features are beneficial for improved high resolution bioimaging. In this study, compound A-ONOO was screened, to determine whether the energy level difference can be specifically modulated by peroxynitrite (ONOO^−^) and environmental viscosity. The chemically stable coumarin was used as the two-photon energy donor and linked by alkyne bonds to an acceptor to develop a mitochondria-targeting two-photon TBET fluorescent probe Mito-ONOO (Scheme 1). The experimental results indicated that the probe accurately monitors the real-time oxidative stress and cellular metabolism of autophagy during a stroke. In addition, we found that during cellular oxygen-glucose deprivation/reoxygenation, cells produce ONOO^−^, causing cellular oxidative stress and significant cellular autophagy after 15 min. These results provide vital information that could be used for stroke diagnosis, treatment, and drug design.

## Results and discussion

### Rational design of probe Mito-ONOO

To accurately monitor the real-time oxidative stress and cellular autophagy during stroke, a probe requires precise mitochondria-anchoring capability, high fidelity and rapid response to cellular reactive oxygen species (ROS, oxidative stress could increase the level of mitochondrial ONOO^−^) and viscosity (autophagy could reduce the viscosity of mitochondria).^17^ To date, reactive oxygen species (ROS)-sensitive probes are commonly used to indicate cellular oxidative stress.^18^ However, the reaction activity of biological ROS varies, resulting in a wide variation in the reaction time between different ROS and the probes, and ultra-fast responses are essential to monitor oxidative stress in real-time. Current fluorescent probes typically exhibit very fast response (<1 min) to highly reactive ROS such as hypochlorous acid (HOCl), hydroxyl radicals (˙OH), and peroxynitrite (ONOO^−^).^19–24^ However, the same oxidative capacity of these ROS makes it difficult for them to be distinguished between. Therefore, we first synthesized 15 near-infrared (NIR) fluorophores with mitochondria-targeting unit and screened them using H_2_O_2_, GSH, H_2_S, SO_2_, NO, ONOO^−^, HClO, and ˙OH as shown in Fig. S1.† The fluorescence response of the 15 compounds and common reactive substances was evaluated, and the results are given in Fig. S2b.† Compounds a7, a8, a10, and a12 are chemically stable and do not respond to any of the analytes, compounds a2, a3, a4, a5, a6, a9, a11, and a14 do not exhibit a specific response to a single analyte, and compounds a1, a13, and a15 respond specifically to ONOO^−^. In particular, compound a1 exhibits a significantly higher response than compounds a13 and a15. Compound a1 was therefore selected as the energy acceptor and renamed as A-ONOO. The proposed response mechanism is shown in Fig. S2a.† The ethylene bond is oxidized to the aldehyde group by ONOO^−^, and the conjugated structure and intramolecular charge transfer of the molecule is significantly altered, resulting in changes in the absorption and fluorescence emission (Fig. S2c–e†). It should be noted that the turn off response of A-ONOO to ONOO^−^ is different from previously reported ONOO^−^ selective fluorescent probes. Due to the high oxidation strength of ONOO^−^, it is reasonable to expect that any fluorophore generated (the product of the reaction between the probe and ONOO^−^) during detection will be oxidized during the recognition process, resulting in a disappearance of fluorescence. This phenomenon can occur, when significant amounts of ONOO^−^ are present in the system, resulting in weak fluorescence. In addition, this kind of probe usually displays a very short operational range, which is also not conducive for the detection of ONOO^−^. Importantly, for A-ONOO the product is non fluorescent and as such the detection signal will not be affected, which is important to help improve the reliability of the monitoring system. Since the product 4-dimethylaminobenzaldehyde after the reaction of A-ONOO with ONOO^−^ is non fluorescent, additional ONOO^−^ cannot continue to influence the fluorescence output. Therefore, A-ONOO was chosen as the energy acceptor, and combined using an alkyne with coumarin as a two-photon fluorophore energy donor to generate the TBET system (Mito-ONOO). The advantage of our strategy is that the probe reacts with ONOO^−^ with high specificity. Then after reacting with ONOO^−^, the signal of the energy donor coumarin is released, which was not affected by any interfering species including excess ONOO^−^, thus enabling high-fidelity detection of ONOO^−^ in complex biological environments. Importantly, the fluorescence peaks before and after reaction between the probe and ONOO^−^ are separated in the TBET system (which does not require spectral overlap between the emission of the energy donor and the absorption of the energy acceptor, but only requires the energy level of the energy donor to be higher than that of the energy acceptor^16^), which is significant for improving the sensitivity and resolution of the probe (Scheme 1). The detailed synthetic procedure and characterization data for the energy donor 1, acceptor A-ONOO, and probe Mito-ONOO are described in Scheme S1, S2, and Fig. S24–S35.† In addition, the products were confirmed using high-resolution mass spectrometry (HRMS), high performance liquid chromatography (HPLC) and nuclear magnetic resonance spectroscopy (1H NMR). As shown in Fig. S3 and S4,† when Mito-ONOO was reacted with ONOO^−^ a mass peak at *m*/*z* = 491.26132, representing the reaction product, was observed (Fig. S3†). The reaction of the probe Mito-ONOO with ONOO^−^ was further confirmed using ^1^H NMR spectroscopy. As shown in Fig. S4c,† the chemical shift of the methyl hydrogen (H_b_) of the pyridine salt of the probe was 4.8 ppm. However, when 10 eq. of ONOO^−^ was added, the chemical shift changes to 10.3 ppm (aldehyde hydrogen H_a_), indicating that product 6 was formed (Fig. S4a†). When the amount of ONOO^−^ was increased further to 20 eq., H_b_ disappears, indicating that the reaction is complete. Therefore, these experiments confirmed the oxidation reaction of probe Mito-ONOO with ONOO^−^. The carbon–carbon double bond was broken, and the aldehyde group was generated to obtain product 6.

### Validation of the TBET “on-off” mechanism

**Fig. 1:**
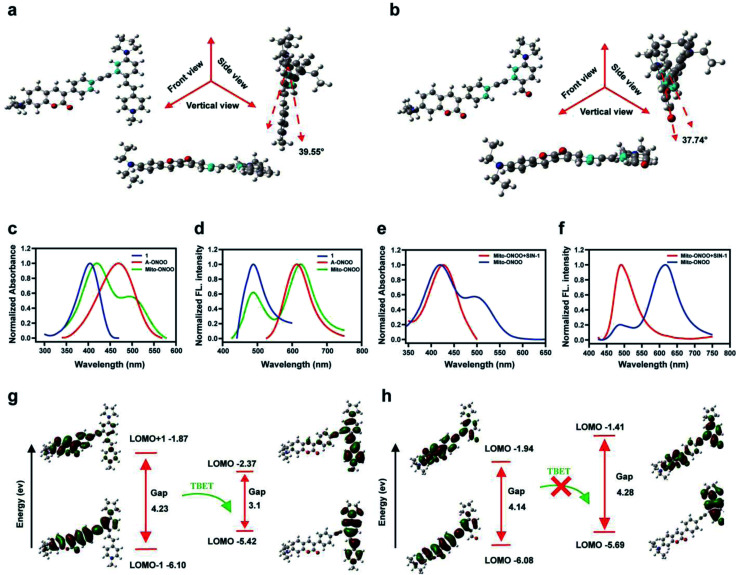
Multiview images (front view, side view and vertical view) of rotatable dihedral angle of (a) Mito-ONOO and (b) 6 that linked the energy donor and acceptor; (c) normalized absorption and (d) fluorescence emission spectra of the donor 1 (black line; *λ*_ex_ = 416 nm), acceptor A-ONOO (red line; *λ*_ex_ = 476 nm) and the probe Mito-ONOO (blue line; *λ*_ex_ = 416 nm). (e) Normalized absorption and (f) normalized fluorescence emission of Mito-ONOO (black line; *λ*_ex_ = 416 nm) and the product 6 (red line, *λ*_ex_ = 416 nm). Frontier orbital ordering estimated by DFT calculations for (g) Mito-ONOO and (h) 6. In (d) and (f), *λ*_ex_ = 416 nm.

Using Gaussian 09 (DFT in B3LYP/6-31G (d) level), we evaluated the frontier orbitals of Mito-ONOO and the product 6 to confirm the TBET “on-off” process. As shown in Fig. 1a and b, the coumarin plane and alkynyl moiety are non-planar, exhibiting dihedral angles *vs.* the energy acceptors before and after reaction with ONOO^−^ of 39.55° and 37.74°, respectively. This indicates that the conjugation of the alkynyl group between the energy donor and the two acceptors is unfavorable, making the donor and acceptors behave like two independent fluorophores. As shown in Fig. 1c and d, both the absorption and fluorescence emission spectra of Mito-ONOO agree well with that of 1 and A-ONOO, indicating that the energy donor and energy acceptor are independent and that they do not constitute a large conjugated system. These observations indicate that a TBET system was successfully constructed.

Theoretical calculations indicated that for Mito-ONOO, the energy gap of the energy donor was 4.23 ev, and that of the energy acceptor was 3.1 ev, which ensures the occurrence of TBET (Fig. 1g). Such a significant difference of energy gap results in a large pseudo-Stokes shift (distance between the two emission peaks of approximately 130 nm), whereas the energy transfer efficiency is not impacted (approximately 97.5%; Fig. 1d). We then evaluated the changes in absorption and fluorescence spectrum of Mito-ONOO with the addition of ONOO^−^. The results indicated that the absorption of the probe at 400 nm before and after reaction with ONOO^−^ did not change significantly, while the peak at 500 nm disappeared, indicating that the structure of 1 before and after the reaction of Mito-ONOO with ONOO^−^ did not change, however A-ONOO was destroyed (Fig. 1e). Similarly, the fluorescence emission peak at 621 nm disappeared after reaction of Mito-ONOO with ONOO^−^, and the emission peak at 495 nm was significantly enhanced, indicating an “on-off” TBET process was occurring (Fig. 1f). As such, simultaneous enhancement of the ratiometric signal change and separation of the dual emission peaks was ensured, which enhances the sensitivity and resolution of sensing. In contrast, the energy gap of product 6 within the energy donor moiety only exhibited a slight change (4.14 ev), whereas that of the energy acceptor moiety increased to 4.28 ev (Fig. 1h). This indicates that the TBET process was blocked by 6. These findings confirm that ONOO^−^ is detected according to a TBET “on-off” mechanism and indicates the successful construction of an ONOO^−^ probe using this strategy.

### Fluorometric detection of ONOO^−^ using Mito-ONOO

**Fig. 2:**
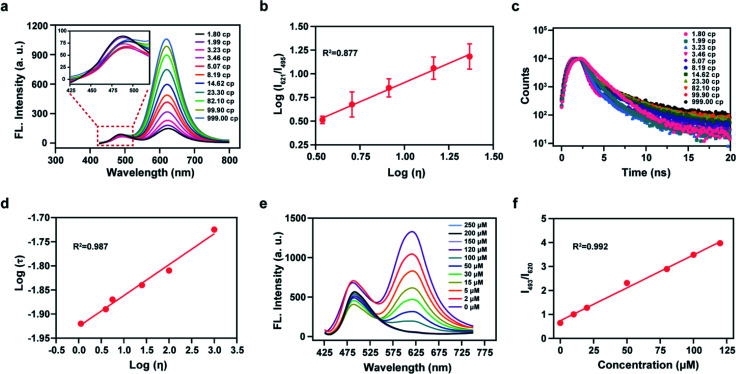
(a) Fluorescence spectra of Mito-ONOO (10 μM) at different solution viscosities in an EtOH/glycerol system *λ*_ex_ = 416 nm; (b) linear relationship between the ratio of log *I*_621_ and *I*_495_*vs.* log *η*; (c) fluorescence lifetime spectra of Mito-ONOO (10 μM) at different solution viscosities in EtOH/glycerol system; (d) linear relationship between log *τ* and log *η*. (e) fluorescence spectra of Mito-ONOO (10 μM) in the presence of increasing SIN-1 concentration (0, 2, 5, 15, 30, 50, 100, 120, 150, 200 and 250 μM) *λ*_ex_ = 416 nm; (f) calibration curve for the determination of SIN-1 in PBS buffer (10 mM, pH 7.4, containing 30% EtOH and 40% glycerol). Data represent the mean of three replicates and the error bars indicate the SD.

We then analyzed the photophysical properties of the energy donor 1 to determine appropriate *in vitro* detection conditions. The absorption and fluorescence spectra of 1 are given in Fig. S5a and S5b,† respectively, and the photophysical parameters are given in Table S1.† Resulting from a D–π–A structure, the absorption spectra of 1 exhibited an obvious bathochromic shift with an increase of solvent polarity (Fig. S5a†). Meanwhile, the fluorescence emission intensity varied in solvents with different polarities (Fig. S5b†). These results indicated that 1 exhibits an absorption maximum (*λ*_abs_) at 399 nm (*ε* = 0.68 × 10^4^ M^−1^ cm^−1^) and a fluorescence maximum (*λ*_fl_) at 482 nm (*Φ* = 0.83, in which *Φ* is the quantum yield) in ethanol (Fig. S5c†). Considering the sensitivity of detection and to simulate a biological environment, we chose a PBS (10 mM, pH 7.4, 0.9% NaCl, containing 40% EtOH) as our subsequent detection medium. Under these conditions the maximal TP action cross-section (*δΦ*, in which *δ* is the TP absorption cross-section) value of 1 was determined to be 120 GM at 810 nm (Fig. S5d†). We then linked 1 and A-ONOO using an acetylenic bond to form the TBET system, which permits a large separation of the two emission peaks. We then evaluated the solubility of the probe at a concentration of 10 μM in PBS buffer (containing 1% EtOH), for application in cell imaging experiments (Fig. S5e and S5f†). Sadly, in the presence of varying concentrations (0, 50, 100 and 200, μM) of SIN-1 (acting as the source of ONOO^−^), the long-wavelength fluorescence signal of Mito-ONOO was weak, and the response to ONOO^−^ was not significant (Fig. S6†). We suspected that the torsion of the *σ* bond of the energy acceptor results in a low *Φ* in PBS. However, we found that the probe exhibits a good response to viscosity. Therefore, the optical performance in an ethanol-glycerol system was evaluated, and the results are given in Fig. 2a and b. The maximal emission of Mito-ONOO at 490 nm was slightly weakened as the viscosity increased. While, as the glycerol fraction was increased from 0 to 100%, the fluorescence intensity of Mito-ONOO at 621 nm was remarkably increased (Fig. 2a). Moreover, the fluorescence intensity ratio exhibited a good linear relationship between log (*I*_621_)/log (*I*_495_) and log (*I*_viscosity_) in the range of 1.56–25 cP (Fig. 2b). This viscosity-dependent fluorescence changes confirm a twisted intramolecular charge transfer process in the energy acceptor system. While the fluorescence lifetime of Mito-ONOO at different viscosity exhibited a similar variation (Fig. 2c), and linear relationship between log *τ* and log *η* in accordance with the variational Förster–Hoffmann equation, as shown in Fig. 2d (in which *τ* is the fluorescence lifetime and *η* is viscosity). Fig. 2e displays the response of the probe towards ONOO^−^ in PBS buffer (10 mM, pH 7.4, containing 30% EtOH and 40% glycerol), and indicates that upon the addition of SIN-1, the fluorescence emission at 621 nm decreases and the fluorescence intensity at 495 nm significantly increases. The ratiometric signal (*I*_495_/*I*_621_, also termed *F*_green_/*F*_red_) exhibits a good linear relationship over a concentration range of 63.4 nM–125 μM (Fig. S7c† and Fig. 2f). In the presence of 150 μM SIN-1, the fluorescence signal of the reaction system reached a plateau in approximately 1 min, and a 4.7-fold enhancement of the ratiometric signal was achieved (Fig. S7a†). The detection limit was calculated to be 63.4 nM according to the 3*s*_b_/*m* criterion, where *m* is the slope of the calibration curve and *s*_b_ is the standard deviation of the blank (*n* = 11). To determine the specificity of Mito-ONOO, we then evaluated the response towards a series of potential interfering species. As depicted in Fig. S7b,† compared to the significant signal enhancement observed for ONOO^−^, the potential interfering species (including positive ion, reactive species, acids, alkalis, enzymes and proteins) resulted in negligible changes to the ratiometric fluorescence signal. Fig. S7e† indicates that Mito-ONOO enhances the ratiometric signal (*I*_621_/*I*_495_) in the presence of proteins, while the hydrolysis of many mismatched proteins in the mitochondria during autophagy leads to a decrease in mitochondrial viscosity. The above results confirm that the probe can monitor *in situ* mitochondrial autophagy (mitophagy). To ensure that aggregation of Mito-ONOO does not hinder reactivity with SIN-1, dynamic light scattering (DLS) was used to evaluate the different nano-aggregates formed. In Fig. S8,† regardless of the presence or absence of glycerol, the DLS signals of Mito-ONOO was less than 100 nm, which was attributed to the positive charge of Mito-ONOO, which increases the solubility of the probe in water and ensures good reactivity with SIN-1. Significantly, the larger particle size of product 6 after reaction with SIN-1 does not affect the reactivity of Mito-ONOO with SIN-1. The above experiments indicate that Mito-ONOO reactivity with SIN-1 is probably not affected by aggregation. To fully understand the selectivity and chemical stability of the probe for ONOO^−^ in complex biological environments, we then evaluated the response of the probe to different interferents (H_2_O_2_, O_2_^˙−^, H_2_S, SO_3_^2−^, NO, HOCl and ˙OH) at different concentrations (0, 1, 2, 5, 8, 10, 15, 20, 30, 50 and 100 μM), as shown in Fig. S9.† The results indicated that no significant changes in the fluorescence ratio for the interfering species and the probe. We then evaluated the response of the fluorophore (the product of the probe to ONOO^−^ reaction) with different interfering species (H_2_O_2_, O_2_^˙−^, H_2_S, SO_3_^2−^, NO, HOCl, ˙OH and ONOO^−^) at a concentration of 100 μM. The signal ratio was found to remain unchanged (Fig. S10†), indicating that the fluorophore has strong chemical stability and can effectively resist interference by active substances in complex biological environments, thus effectively avoiding false-negative results. The effect of pH on the response of Mito-ONOO to SIN-1 was also evaluated (Fig. S7d†). The results confirmed that Mito-ONOO produced the largest signal upon reaction with SIN-1 over a pH range of 4.2–8.0, and the fluorescence of the product was stable over the physiological pH range.

### Cell imaging

We then evaluated Mito-ONOO for determining changes of ONOO^−^ levels in live cells. Prior to this evaluation, the cytotoxicity of Mito-ONOO was determined using a standard 3-(4,5-dimethylthiazol-2-yl)-2,5-diphenyltetrazolium bromide (MTT) assay, and the results confirmed that Mito-ONOO exhibited low cytotoxicity towards BV-2 cells (Fig. S11†). In addition, the fluorescence intensity of the green channel and red channel remained almost unchanged after the probe-loaded cells (10 μM) were irradiated with 810 nm two-photon excitation for 60 min (Fig. S12†). These results confirm that Mito-ONOO exhibits good biocompatibility and photostability and was suitable for real-time monitoring and analysis of the biological processes of live cells. Subsequently, the mitochondrial localization of Mito-ONOO was evaluated using a multicolor localization assay. A commercially available mitochondrial tracker, Mito-Tracker-Red, was used as a reference to check the location of Mito-ONOO in BV-2 cells. The images were collected using both the green (*λ*_ex_ = 810 nm, *λ*_em_ = 450–530 nm) and red channels (*λ*_ex_ = 560 nm, *λ*_em_ = 595–650 nm). As shown in Fig. S13,† the signal of Mito-ONOO in the green channel overlaid extremely well with the fluorescence of Mito-Tracker-Red in the red channel. Pearson's co-localization coefficient between the signals in the green and red channels was calculated to be 0.98, confirming that Mito-ONOO has good mitochondrial targeting efficiency in living cells, providing a basis for the detection of mitochondrial ONOO^−^ and autophagy.

The ability of Mito-ONOO for monitoring fluctuations in the viscosity of live cells was then determined by adding dexamethasone (5 μM, inducer of cellular viscosity)^14^ and changing the temperature (intracellular viscosity increases with a decrease of temperature). Using two-photon excitation (810 nm), the fluorescence of the red channel (*F*_red_, emission range 600–700 nm) enhanced, while the fluorescence of green channel (*F*_green_, emission range 450–530 nm) weakened, and the ratio signal *F*_green_/*F*_red_ gradually decreased with a decrease of temperature and the addition of dexamethasone (Fig. S14†). These results indicate that Mito-ONOO could be used to detect viscosity changes of living cells. Moreover, through the addition of SIN-1 (3 eq.) and dexamethasone it was observed that *F*_red_ almost disappeared, while *F*_green_/*F*_red_ was higher than that of the 37 °C and the control group, which further confirmed that the probe could achieve a simultaneous response towards viscosity and ONOO^−^. Next, the utility of Mito-ONOO for the ratiometric fluorescence detection of exogenous ONOO^−^ in living cells were examined. BV-2 cells loaded with Mito-ONOO and ionophores (monensin and nystatin, inducers of cell viscosity) exhibited negligible fluorescence in the green channel and strong fluorescence in the red channel (Fig. S15†). Then for cells treated with SIN-1 a dramatic decrease in the red channel fluorescence and remarkable enhancement of the green channel fluorescence was observed. To eliminate latent interference induced by other biological relevant ROS and reactive sulfur species (RSS), the selectivity of Mito-ONOO for visualizing ONOO^−^ was then validated in BV-2 cells. HOCl and SO_3_^2−^ were selected as representative interfering ROS and RSS, respectively, and the production of exogenous ONOO^−^ was stimulated by SIN-1. As shown in Fig. S16,† BV-2 cells incubated with ionophores together with Mito-ONOO and different interferents (HOCl and SO_3_^2−^) exhibited negligible changes in fluorescence compared with the control group. However, the cells exhibited strong fluorescence changes in both channels when pre-treated with SIN-1 before incubation with Mito-ONOO (Fig. S16†), while the signal ratio weakened after incubation with minocycline (a scavenger of ONOO^−^), indicating that the increased *F*_green_/*F*_red_ was mainly attributable to reaction between the probe and the generated ONOO^−^. Furthermore, SIN-1 usually produces ONOO^−^ by releasing NO and O_2_^˙−^ simultaneously. Therefore to exclude the effect of NO and O_2_^˙−^ on the fluorescence intensity, AG (NO scavenger) and TEMPO (an O_2_^˙−^ scavenger) were added. The results indicated that the *F*_green_/*F*_red_ of cells stained with Mito-ONOO remained unchanged, and that Mito-ONOO could specifically detect and image ONOO^−^. Rapamycin and lipopolysaccharide (LPS) were used to induce an autophagy and inflammatory response in BV-2 cells, respectively.^25^ The results indicate a slight increase in the fluorescence intensity of the green channel after transfection of rapamycin and LPS-treated BV-2 cells with the probe Mito-ONOO compared to the control group, while a significant weakening of the fluorescence intensity of the red channel and a significant enhancement of *F*_green_/*F*_red_ occurs (Fig. S17†). More importantly, the decrease of the fluorescence intensity of the red channel of the LPS group was higher than that for the rapamycin group, indicating that multiple metabolic processes control the Mito-ONOO fluorescence signal. In addition, *F*_green_/*F*_red_ of both drug-induced groups were reduced when minocycline and 3-methyladenine (3-MA) were used to scavenge ONOO^−^ and inhibit cellular autophagy, respectively, but remained higher than in the control group, indicating that inflammation could simultaneously induce the production of ONOO^−^ and cellular autophagy.

### Fluorescence imaging of oxidative stress/autophagy during OGD/R

**Fig. 3:**
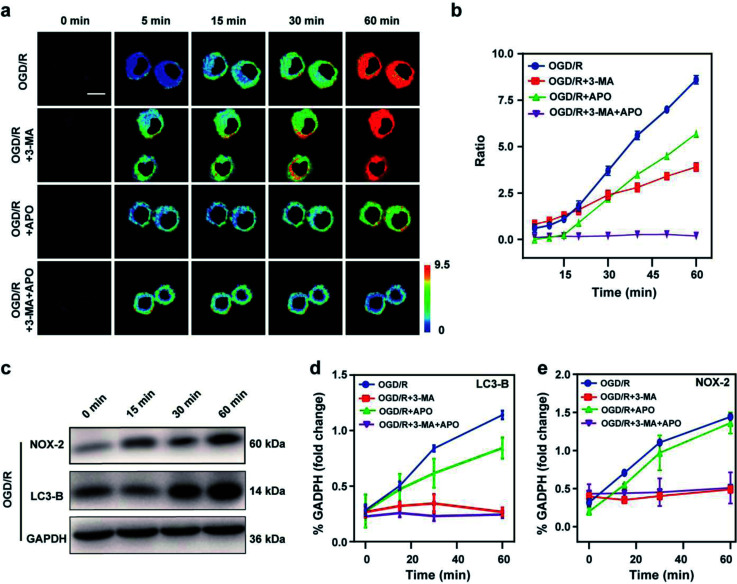
Two-photon fluorescence imaging (a) and time course of fluorescence ratio changes (b) of BV2 cells. Cells incubated with 10 μM Mito-ONOO during OGD/R with the first line: control; the second line: APO; the third line: 3-MA and the fourth line: APO+3-MA. For the fluorescent images, the experiment was repeated using three cultures; similar results were obtained each time. Scale bar, 20 μm. For the time course of fluorescence ratio changes, data are presented as the mean ± SD (control: *n* = 40 cells from three cultures; APO: *n* = 37 cells from three cultures; 3-MA: *n* = 33 cells from three cultures; APO+3-MA: *n* = 46 cells from three cultures). (c) Western blotting illustrates the expression of NOX-2, LC3-B and GAPDH in BV-2 cells during OGD/R and pre-treated with APO, 3-MA, and APO+3-MA at different times (0, 15, 30 and 60 min). (d and e) Quantification data of the western blot results of LC3-B (d) and NOX-2 (e) at different time points. *λ*_ex_ = 810 nm; *λ*_em_ = 450–530 nm (the green channel) and 600–700 nm (red channel). In (d) and (e), the error bars indicate the SD.

**Fig. 4:**
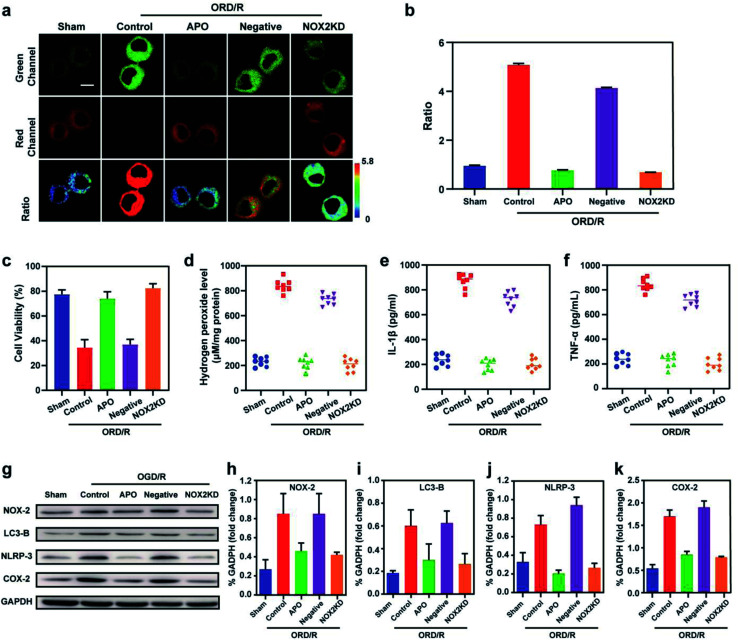
Two-photon fluorescence imaging (a) and averaged fluorescence ratio changes (b) of Mito-ONOO-loaded (10 μM) BV-2 cells undergoing OGD/R when subjected to different treatments: sham group (untreated cells); control group (OGD/R treated cells); APO group (APO treated cells during OGD/R), negative group (negative control group of NOX2KD) and NOX2 KD group (NADPH oxidase 2 gene knockout). For the fluorescent images, the experiment was repeated using three cultures; similar results were obtained each time. Scale bar, 20 μm. For the fluorescence ratio change: for untreated cells, sham group: *n* = 48 cells from three cultures; for OGD/R treated cells, control group: *n* = 61 cells from three cultures; for APO treated cells during OGD/R, APO group: *n* = 51 cells from three cultures; for negative control group of NOX2KD, negative group: *n* = 65 cells from three cultures; for NADPH oxidase 2 gene knockout, NOX2KD group: *n* = 55 cells from three cultures. (c) CCK-8 analysis changes in cells from samples in (a). (d) H_2_O_2_ levels in OGD/R group. (e and f) ELISA assay of (e) IL-1β and (f) TNF-α levels in OGD/R group. (g) Western blotting illustrates the expression of NOX-2, LC3-II, NLRP-3, COX-2 and GAPDH in BV-2 cells of (a). (h–k) Quantification data of the western blot results of NOX-2 (h), LC3-B (i), NLRP-3 (J) and COX-2 (k) in BV-2 cells of (a). *λ*_ex_ = 810 nm; *λ*_em_ = 450–530 nm (the green channel) and 600–700 nm (red channel). In (c) and (h–k), the error bars indicate the SD.

Stroke is an acute cerebrovascular disease that seriously threatens human health. Previous research has indicated that a sequence of biological events occurs during stroke, including cellular oxidative stress and cellular autophagy. Inspired by the previous results, we inferred that Mito-ONOO could act as indicator for real-time tracking of cellular oxidative stress and autophagy processes, simultaneously. Therefore, an oxygen-glucose deprivation (OGD) model was constructed, and during reoxygenation (OGD/R, which was used to simulate the process of cell ischemia/reperfusion during stroke. Briefly, cells were kept in sugar-free DMEM and a three-gas incubator for 12 hours without oxygen, and then the normal state of cultured cells was resumed) were tracked in real time using Mito-ONOO.^26–28^ As shown in Fig. 3a and S18,† the fluorescence intensity ratio of the OGD/R cells under different treatment conditions (including control group, APO group, 3-MA group and APO+3-MA group) increased gradually over the first 5 minutes. This phenomenon indicates that the probe has fully entered the cell after 5 minutes. For the next 10 minutes (5–15 min), the ratio intensity of OGD/R and OGD/R+3-MA groups showed a significant linear increase, while the other two groups did not change significantly, indicating that ONOO^−^ was produced in the first 15 minutes of cell reperfusion. In addition, apocynin (APO, NADPH oxidase 2 inhibitor)^29^ had the ability to inhibit NADPH oxidase 2 (NOX2, one of the main sources of ROS in the central nervous system),^30^ thereby inhibiting the production of ONOO^−^ by reducing the level of cellular oxidative stress, which resulted in no change in the ratio intensity of OGD/R+APO and OGD/R+APO+3-MA. Furthermore, 15 min after the occurrence of OGD/R in cells, the trend in the ratio intensity of OGD/R+3-MA and OGD/R+APO+3-MA groups still maintained a good linear relationship with that before 15 min, while OGD/R and OGD/R+APO groups exhibited a significant increase in the linear trend compared to 15 min before (the slope of the linear curve increases), and an obvious inflection point appears at 15 min (Fig. 3c). We believe that this inflection point is because the model OGD/R cells first undergo oxidative stress then autophagy begins at 15 min. Since 3-MA can inhibit the occurrence of autophagy,^31^ the ratio intensity of OGD/R+3-MA and OGD/R+APO+3-MA groups failed to have an inflection point. Western blot analysis confirmed this hypothesis. The results indicated that cells exhibited significant expression of NOX-2 over the first 15 min of OGD/R (Fig. 3c and e). Then, after 15 min, the amount of LC3-B (cellular autophagy-specific expression protein^32,33^) increased significantly (Fig. 3c and d). To further prove the origin of the inflection point, we used the same model as the confocal studies (Fig. 3d, e and Fig. S19†). The results indicated that the expression of NOX-2 in the reperfusion OGD/R+APO and OGD/R+APO+3-MA groups remained almost unchanged, while the OGD/R+3-MA group showed the same trend of change as OGD/R. However, the expression of LC3-B did not significantly change 15 min before reperfusion. After 15 min, the OGD/R+APO group showed the same trend as OGD/R, while no significant change of the OGD/R+3-MA and OGD/R+APO+3-MA group was observed. The above experiment once again shows that our hypothesis is valid. To further understand the relationship between OGD/R, cellular oxidative stress and autophagy, pharmacological inhibition (apocynin, APO) and gene knockout (NOX-2 KD) were used to inhibit NOX-2 protein expression, indicating that the OGD/R process (oxygen-glucose deprivation 12 h and reoxygenation 1 h) could lead to NOX-2 protein overexpression, thereby causing the cells to undergo oxidative stress and induce cellular autophagy. As shown in Fig. 4a and b, two-photon fluorescent images indicated that *F*_green_/*F*_red_ was significantly lower in both APO and NOX-2KD groups than the OGD/R group, while *F*_green_/*F*_red_ is significantly higher in the negative control group (negative control group of NOX-2 KD) compared to the NOX-2 KD group. Thus, the experiments confirmed that NOX-2 protein could regulate the oxidative stress state and cellular autophagy. Evaluation of cell viability using a CCK-8 assay indicated that the cellular damage caused by the OGD/R process could be effectively improved by inhibiting NOX-2 expression (Fig. 4c), illustrating that this method could be applied for the treatment of cerebral ischemia/reperfusion injury. Moreover, the levels of inflammatory factors TNF-α, IL-1β and hydrogen peroxide (H_2_O_2_) elevated when autophagy occurred (Fig. 4d–f), confirming the relevance of autophagy to the ROS level and inflammation. Western blot analysis confirmed the accuracy of our approach (Fig. 4g–k). While Mito-ONOO is unable to directly distinguish between autophagy (a decrease of mitochondrial viscosity) and oxidative stress (an increase of ONOO^−^ levels). It can be used to track the occurrence of autophagy and oxidative stress in the process of OGD/R. In addition, we could use Mito-ONOO to determine that oxidative stress precedes autophagy and can activate autophagy.

### *In vivo* imaging of Mito-ONOO during stroke

**Fig. 5:**
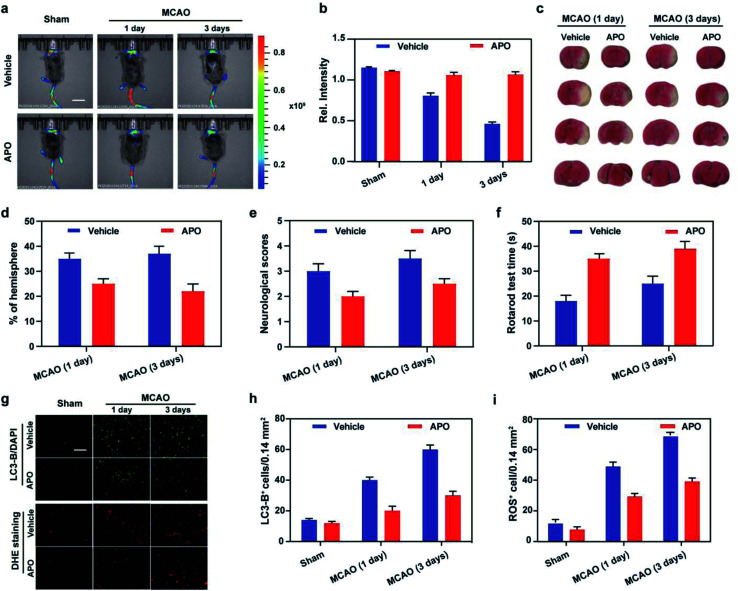
a) *In vivo* imaging of ONOO^−^ outburst and autophagy in brain undergoing middle cerebral artery occlusion (MCAO) at different time subjected to different treatments (Mito-ONOO: 200 μL, 200 μM): sham group (mice not undergoing MCAO); MCAO group (mice undergoing MCAO); vehicle group (injection of saline to mice tail veins). APO group (injection of apocynin to mice tail veins). Scale bar = 2 cm. *λ*_ex_ = 530 nm; *λ*_em_ = 600–700 nm. (b) Histogram of the average fluorescent intensities in panel (a). (c) TTC staining images of infarct regions in the ipsilateral hemisphere slices undergoing different treatments. (d) The percentages of infarct size to contralateral hemisphere of panel (c). (e) Neurological scores. (f) Rotarod test. (g) Expression of LC3B revealed by immunofluorescent staining and ROS revealed by DHE staining from samples in (a). (h) Quantification of LC3-B-positive cells in the ischemic border. (i) Quantification of ROS-positive cells in the ischemic border. *n* = 5 per group. Scale bar = 50 μm. In (b), (d–f), (h) and (i), the error bars indicate the SD.

Based on the results obtained using cellular OGD/R models, we endeavored to use Mito-ONOO to monitor ONOO^−^ and autophagy fluctuations during the process of stroke *in vivo*. Initially, the *in vivo* cytotoxicity of Mito-ONOO (200 μM, 200 μL) was examined *via* hematoxylin and eosin (H&E) staining of organs after intravenous injection of the probe. The images of all tissues exhibited no noticeable signs of organ damage after injecting the probe (Fig. S20†), implying the negligible toxicity and side effects of Mito-ONOO in the mouse. Using the z-scan mode of the two-photon microscope, we then investigated the distribution of Mito-ONOO at different depths of mouse brain tissue (Fig. S21†). The accumulated images revealed that Mito-ONOO can homogeneously stain the tissues and that it can be visualized at depths of up to 220 μm in mouse brain tissues under TP excitation. With the above results in hand, we clearly observed that NOX-2 protein overexpression was upregulated during stroke *in vivo*, which can cause oxidative stress of brain tissue and results in cellular autophagy. As shown in Fig. 5, a mouse stroke model was successfully constructed using middle cerebral artery occlusion (MCAO)^34^ combined with 2,3,5-triphenyltetrazolium chloride (TTC, measures tissue viability used to evaluate infarct size) staining (Fig. S5c† and 5d). The fluorescence intensity of the brain tissue sites was significantly reduced on day 1 and day 3 after stroke in mice using *in situ* imaging (Fig. 5a and b). The fluorescence intensity of APO-treated (2.5 mg kg^−1^) mice was not significantly different from the sham group (mice not undergoing MCAO), indicating that NOX-2 protein was a key factor leading to oxidative stress in tissues and causing cellular autophagy (Fig. 5a and b). The slicing experiments confirmed the conclusions from both cellular and *in vivo* experiments. The enhanced fluorescence signal indicated that the probe could cross the blood–brain barrier and enter the mouse brain tissue (Fig. S22†). While TTC staining and behavioral assays indicated that infarct volumes, neurological scores and rotarod test were significantly reduced in APO mice compared with those that were simply injected with saline 1 and 3 days after perfusion (Fig. 5c–f). In addition, we evaluated the ROS synthesis protein (NOX-2), autophagy related protein (Beclin-1, LC3-B) and inflammatory factors (NLRP3 and IL-1β) expression and ROS levels using western blot and dihydroethidium (DHE, a ROS fluorescent probe that can be oxidized by ROS to form ethidium oxide) staining, respectively (Fig. 5g–i and S23†). The result indicated that the production of proteins and ROS were markedly increased after 3 days of stroke. Furthermore, APO injection significantly suppressed the production of ROS and of those proteins, indicating that the stroke process was accompanied by neuroinflammation.

## Conclusions

A viscosity and ONOO^−^ bifunctional two-photon ratiometric fluorescent probe (Mito-ONOO) was developed based on the TBET mechanism to track autophagy and oxidative stress during OGD/R in real-time. The probe exhibited high fidelity for imaging viscosity and ONOO^−^, mitochondrial targeting capability, and baseline separation of the dual emission peaks, facilitating the real-time and *in situ* tracking of autophagy and oxidative stress in living cells and *in vivo* with high resolution and reliability. Mito-ONOO is the first probe to enable real-time visualization of autophagy and oxidative stress during OGD/R using ratiometric analysis. Our results indicated that cells produced ONOO^−^, leading to cellular oxidative stress during cellular OGD/R. Then after 15 min, there was a significant onset of cellular autophagy. We anticipate that these results will provide the impetus to develop new practical systems for stroke diagnosis, treatment, and drug design.

## Data availability

All data supporting this study are provided as ESI† accompanying this paper.

## Author contributions

Wei Hu: conceptualization, investigation, methodology, data curation, writing – original draft. Taotao Qiang: conceptualization, supervision, validation, funding acquisition. Li Chai: validation, data curation. Tianyu Liang: methodology, data curation. Longfang Ren: validation, supervision. Chunya Li: supervision, validation, funding acquisition. Tony D. James: conceptualization, validation, supervision, project administration, writing – review and editing.

## Conflicts of interest

TDJ acts as an academic consultant for TQ as part of a guest professorship at SUST.

## Supplementary Material

SC-013-D1SC06805A-s001

## References

[cit1] Bruggeman F. J., Westerhoff H. V. (2007). Trends Biotechnol..

[cit2] Kell D. B. (2004). Curr. Opin. Microbiol..

[cit3] Nicholson J., Lindon J. (2008). Nature.

[cit4] Ying W. H. (2008). Antioxid. Redox Signaling.

[cit5] Trachootham D., Alexandre J., Huang P. (2009). Nat. Rev. Drug Discovery.

[cit6] Weinberg S. E., Chandel N. S. (2015). Nat. Chem. Biol..

[cit7] Spitaler M. M., Graier W. F. (2002). Diabetologia.

[cit8] Zhang S., Liu X., Bawa-Khalfe T., Lu L.-S., Lyu Y. L., Liu L. F., Yeh E. T. H. (2012). Nat. Med..

[cit9] Mayevsky A., Rogatsky G. G. (2007). Am. J. Physiol. Cell Physiol..

[cit10] Nicholson J. K., Wilson I. D. (2003). Nat. Rev. Drug Discovery.

[cit11] Huang Y. F., Zhang Y. B., Huo F. J., Chao J. B., Cheng F. Q., Yin C. X. (2020). J. Am. Chem. Soc..

[cit12] liang T. Y., Qiang T. T., Ren L. F., Cheng F., Wang B. S., Li M. L., Hu W., James T. D. (2022). Chem. Sci..

[cit13] Cheng F., Qiang T. T., Ren L. F., Liang T. Y., Gao X. Y., Wang B. S., Hu W. (2021). Analyst.

[cit14] Liang T., Zhang D., Hu W., Tian C., Zeng L., Wu T., Lei D., Qiang T., Yang X., Sun X. (2021). Talanta.

[cit15] Mao Z. Q., Ye M. T., Hu W., Ye X. X., Wang Y. Y., Zhang H. J., Li C. Y., Liu Z. H. (2018). Chem. Sci..

[cit16] Hu W., Zeng L. Y., Zhai S. Y., Li C. C., Feng W. Q., Feng Y., Liu Z. H. (2020). Biomaterials.

[cit17] Zhang Y. Y., Li Z., Hu W., Liu Z. H. (2019). Anal. Chem..

[cit18] Lou Z. G., Li P., Han K. L. (2015). Acc. Chem. Res..

[cit19] Yuan L., Wang L., Agrawalla B. K., Park S.-J., Zhu H., Sivaraman B., Peng J. J., Xu Q. H., Chang Y.-T. (2015). J. Am. Chem. Soc..

[cit20] Wu D., Chen L. Y., Xu Q. L., Chen X. Q., Yoon J. Y. (2019). Acc. Chem. Res..

[cit21] Guo Q. W., Liu Y. X., Jia Q., Zhang G., Fan H. M., Liu L. D., Zhou J. (2017). Anal. Chem..

[cit22] Zeng L. Y., Xia T., Hu W., Chen S. Y., Chi S. Y., Lei Y. D., Liu Z. H. (2018). Anal. Chem..

[cit23] Lu J., Li Z., Zheng X. R., Tan J. K., Ji Z. Y., Sun Z. W., You J. M. (2020). J. Mater. Chem. B.

[cit24] Wu W. J., Zhang C., Rees T. W., Liao X. X., Yan X., Chen Y., Ji L. N., Chao H. (2020). Anal. Chem..

[cit25] Fu S.-P., Li S.-N., Wang J.-F., Li Y., Xie S.-S., Xue W.-J., Liu H.-M., Huang B.-X., Lv Q.-K., Lei L.-C., Liu G.-W., Wang W., Liu J.-X. (2014). Mediators Inflammation.

[cit26] Wang C., Yang Y.-H., Zhou L., Ding X.-L., Meng Y.-C., Han K. (2020). J. Pharm. Pharmacol..

[cit27] Peng J. L., Wang H. X., Gong Z., Li X. P., He L., Shen Q. Y., Pan J. R., Peng Y. (2020). Mol. Immunol..

[cit28] Yang Z. J., Wang L., Hu Y. J., Wang F. X. (2020). Mol. Med. Rep..

[cit29] Walder C. E., Green S. P., Darbonne W. C., Mathias J., Rae J., Dinauer M. C., Curnutte J. T., Thomas G. R. (1997). Stroke.

[cit30] Neubert M., Ridder D. A., Bargiotas P., Akira S., Schwaninger M. (2011). Cell Death Differ..

[cit31] Hou L. L., Ning P., Feng Y., Ding Y. Q., Bai L., Li L., Yu H. Z., Meng X. M. (2018). Anal. Chem..

[cit32] Wang T. T., Zhu L., Liu H. L., Yu G. Y., Guo Y. L. (2019). Anat. Rec..

[cit33] Chang W. G., Teng J. F. (2015). Int. J. Clin. Exp. Med..

[cit34] Li C. C., Hu W., Wang J. C., Song X. J., Xiong X. X., Liu Z. H. (2020). Analyst.

