# Supplementary material for: Simultaneous tracking of autophagy and oxidative stress during stroke with an ICT-TBET integrated ratiometric two-photon platform
Source: Chem Sci. 2022 Apr 21;13(18):5363–73. doi: 10.1039/d1sc06805a (PMC9093177; doi:10.1039/d1sc06805a)
Supplement: SC-013-D1SC06805A-s001 [file SC-013-D1SC06805A-s001.pdf]

Electronic Supplementary Material (ESI) for Chemical Science.
This journal is © The Royal Society of Chemistry 2022

Simultaneous Tracking Autophagy and Oxidative Stress During Stroke with an ICT-TBET Integrated Ratiometric Two-Photon Platform

Wei Hu,[a] Taotao Qiang,[a,]* Li Chai,[b] Tianyu Liang,[a] Longfang Ren,[a] Fei Cheng,[a] Chunya Li[b,]* and Tony D. James[c,d,]*

[a] *College of Bioresources and Materials Engineering, Shaanxi Collaborative Innovation Center of Industrial Auxiliary Chemistry & Technology, Shaanxi University of Science & Technology, Xi'an, 710021, China.*

[b] *Key Laboratory of Analytical Chemistry of the State Ethnic Affairs Commission, College of Chemistry and Material Science, South-Central University for Nationalities, Wuhan 430074, China.*

[c] *Department of Chemistry, University of Bath, Bath, BA27AY, United Kingdoms.*

[d] *School of Chemistry and Chemical Engineering, Henan Normal University, Xinxiang 453007, China.*

*Corresponding author.

E-mail: t.d.james@bath.ac.uk (Tony D. James), qiangtt515@163.com (Taotao Qiang) and lichychem@mail.scuec.edu.cn. (Chunya Li).

Table of Contents

1. General Information on Materials and Methods 3
Instruments and materials 3
Spectroscopic measurements 3
Determination of the detection limit 4
Quantum yield measurements 4
DFT calculations 4
Water solubility assay 5
Measurement of two-photon cross section 5
Calculation of energy transfer efficiency 5
Viscosity determination and fluorescence measurements 6
Cell culture and imaging 6
Immunofluorescence staining 6
Western blotting assay 7
Cytotoxicity Assay 7
General procedure for detection of H_2O_2, TNF-α and IL-1β concentration 8
Histological staining of the tissue slices 8
OGD/R model 8
Two-photon fluorescence cell imaging 8
Calculation of Mean Fluorescence Intensity 8
Measurement of ROS 9
Middle cerebral artery occlusion (MCAO) model 9
Measurement of infarct volume and neurological deficit 9
Assessment of neurological deficit 10
Rotating beam walking test 10
Rotarod test 10
In vivo imaging studies 10
Statistical analysis 11
2. Synthesis of **Mito-ONOO** and **A-ONOO** 12
3. Supplementary Figures 16
4. Supplementary Table 39
5. References 40
6. ^{1}H NMR Spectra, ^{13}C NMR Spectra, and HRMS Spectra of Compounds 43

1. General Information on Materials and Methods

Instruments and materials

Unless otherwise stated, all solvents and reagents were purchased from commercial suppliers and were used as received without further purification. BV-2 cells were obtained from Procell Life Science & Technology Co., Ltd. 3-(4,5-Dimethylthiazol-2-yl)-2,5-diphenyltetrazolium bromide (MTT), Lipopolysaccharide (LPS), Minocycline, monensin, 2,2,6,6-tetramethylpiperidine-N-oxyl (TEMPO), amino-guanidine (AG), MitoTracker Green FM, Apocynin, Minocycline, 3-Methyladenine and 3-Morpholinosydnonimine hydrochloride (SIN-1) were purchased from Sigma-Aldrich. All aqueous solutions were prepared in ultrapure water with a resistivity of 18.25 MΩ cm (purified by Milli-Q system, Millipore). High-resolution mass spectrometry was performed with LTQ FT Ultra (Thermo Fisher Scientific, America) in MALDI-DHB mode. NMR spectra were recorded on a Bruker-400 spectrometer, using TMS as an internal standard. Response mechanism of probe and $ONOO^-$ was detected using an Agilent 1290 Infinity II/6230 TOF LC-MS system. Absorption spectra were recorded with a UV-vis spectrophotometer (Shimadzu UV-2550, Japan), and one-photon fluorescence spectra were obtained with a fluorimeter (Shimadzu RF-6000, Japan). Two-photon fluorescence spectra were excited by a mode-locked Ti:sapphire femto-second pulsed laser (Chameleon Ultra I, Coherent, America) and recorded with a DCS200PC photon counting with Omno-λ5008 monochromator (Zolix, China). Two photon microscopy was performed on a Zeiss LSM 710 multiphoton laser scanning confocal microscope (Carl Zeiss, Germany). Fluorescence imaging of mice was performed on an IVIS Lumina LT Series III small animal optical *in vivo* imaging system (U.S.A.) with an excitation filter of 500 nm and an emission filter of 650 nm. Experimental mice were anesthetized on an R500IE anesthesia machine. Living Image 4.5 software (PerkinElmer) was used for data analysis.

Spectroscopic measurements

Unless otherwise mentioned, all the measurements using **Mito-ONOO** were evaluated in PBS buffer (10 mM, pH 7.4, containing 30% EtOH and 40% glycerol). After adding SIN-1 and incubating at 37 °C for 5 min, a 500 μL aliquot of the reaction solution was transferred to a quartz cell with an optical length of 1 cm for the measurement of absorbance or fluorescence. The excitation wavelengths were 416 nm and 810 nm, under one-photon and two-photon excitation mode, respectively.

For the selectivity assay, superoxide anion ($O_2^{\bullet-}$) was prepared by dissolving KO_2 in DMSO solution[1]. •OH was generated by Fenton reaction between Fe^{2+} (EDTA) and H_2O_2 quantitatively, and Fe^{2+} (EDTA) concentrations represented •OH concentrations[2]. The $ONOO^-$ source was the donor 3-morpholinosydnonimine hydrochloride (SIN-1, 200 mM)[3]. NO was generated in form of 3-(aminopropyl)-1-hydroxy-3-isopropyl-2-oxo-1-triazene (NOC-5, 100 μM)[4]. H_2O_2 was determined at 240 nm ($\varepsilon_{240\ nm}$ = 43.6 $M^{-1}cm^{-1}$). NO_2 was generated from $NaNO_2$. All the reagents were obtained from Aladdin (USA). All other chemicals were from commercial sources and of analytical reagent grade unless indicated otherwise.

Determination of the detection limit

The limit of detection (LOD) for hydrogen sulfide was calculated based on the following equation:

$$\mathrm{LOD} = 3\sigma/k$$

Where σ represents the standard deviation and k represents the slope of the titration spectra curve among the limited range.

Quantum yield measurements

The measurement of the fluorescence quantum yield was measured by using an ethanol solution of rhodamine B as a standard (10 μM, Φ_r = 0.71) and using the following equation[5].

$$\Phi_s = \frac{A_r \cdot F_s \cdot n_s^2}{A_s \cdot F_r \cdot n_r^2}\, \Phi_r \quad (A \leq 0.05)$$

Where s and r represent the sample to be tested and the reference dye, respectively. A represents the absorbance at the maximum absorption wavelength, F represents the fluorescence spectrum integral at the maximum absorption wavelength excitation, and n represents the refractive index of the sample to be tested or the reference dye solvent.

DFT calculations

To describe the ground state and singlet excited state of TBET system, DFT theoretical calculations were performed[6]. All the calculations were carried out using the Gaussian 09 program package. All the geometries of TBET system were optimized at

B3LYP/6-31+G(d) level using a CPCM solvation model with water as the solvent. The molecular orbital (MO) plots and MO energy levels were computed at the same level of theory.

Water solubility assay

Small amounts of **Mito-ONOO** were dissolved in EtOH to prepare stock solutions (5.0×10^{-2} M). The solution was diluted to ($1.0 \times 10^{-6} \sim 1.0 \times 10^{-4}$ M) in 3.0 mL ultrapure H_2O. In all cases, the concentration of EtOH was maintained at 1%. The plot of fluorescence intensity against the **Mito-ONOO** concentration were linear at low concentration and showed downward curvature at higher concentrations, and the maximum concentration in the linear region was taken as the solubility limit.

Measurement of two-photon cross section

The two-photon cross section (δ) was determined by using reported femto second (fs) fluorescence measurement techniques [7]. **1** and **A-ONOO** was dissolved in PBS buffer (10 mM, pH 7.4, 0.9% NaCl containing 40% EtOH) and the two-photon induced fluorescence intensity was measured at 710-850 nm by using rhodamine B as the reference, whose two-photon property has been well characterized in the literature. The intensities of the two-photon induced fluorescence spectra of the reference and sample emitted at the same excitation wavelength were determined. The two-photon cross section was calculated using $\delta = \delta_r(S_s \cdot \Phi_r \cdot \phi_r \cdot c_r)/(S_r \cdot \Phi_s \cdot \phi_s \cdot c_s)$, where the subscripts s and r stand for the sample and reference molecules, respectively. The intensity of the signal collected by a CCD detector was denoted as S. Φ is the fluorescence quantum yield. ϕ is the overall fluorescence collection efficiency of the experimental apparatus which can be approximated by refractive index of the solvent. The number density of the molecules in solution was denoted as c. δ_r is the two-photon absorption cross section of the reference molecule.

Calculation of energy transfer efficiency

Energy transfer efficiency (E) was calculated using the following equation[8]:

$$E = 1 - F_{DA} / F_D$$

Where, F_{DA} and F_D denote the donor fluorescence intensity with and without an acceptor, respectively.

Viscosity determination and fluorescence measurements

The solvents were obtained by mixing an ethanol-glycerol system in different proportions. Measurements were carried out with an NDJ-8S rotational viscometer, and each viscosity value was recorded[9]. The solutions of **Mito-ONOO** of different viscosity were prepared by adding the stock solution (1.0 mM) 10 μL to 1 mL of solvent mixture (ethanol-glycerol solvent systems) to obtain the final concentration of **Mito-ONOO** (10.0 μM). These solutions were sonicated for 5 minutes to eliminate air bubbles. After standing for 1 hour at a constant temperature, the solutions were measured in a UV spectrophotometer and a fluorescence spectrophotometer.

Cell culture and imaging

BV-2 cells were cultured in Dulbecco's modified Eagle's medium (DMEM, Thermo Scientific) supplemented with 1% penicillin/streptomycin and 10% fetal bovine serum (FBS), and incubated in an atmosphere of 5/95 (v/v) of CO_2/air at 37 °C. Two days before imaging, the cells were passed and placed into glass-bottomed dishes (NEST). For labeling, the cells were washed with serum-free DMEM and then incubated with 10 μM **Mito-ONOO** (containing 1% DMSO) for 30 min at 37 °C.

Immunofluorescence staining

Immunofluorescence was performed as previously described[10]. Ischemic and sham-operated mice were euthanized and perfused with cold PBS, followed by fixation with 4% paraformaldehyde for 2 days. The ischemic brains were cut into 50-μm sections and the free-floating slices were blocked with 0.1 M PBS containing 5% fetal bovine serum and 0.3% Triton X for 1 h at room temperature. After washing, the slices were incubated at 4 °C overnight with the following primary antibodies: anti-LC3B (1:200; ab104224, Abcam, Cambridge, England). The slices were then rinsed and incubated with an Alexa 594-conjugated antibody (1:200; ANT030, Millipore, Billerica, MA) or an Alexa 488-conjugated antibody (1:200; ANT024, Millipore, Billerica, MA for 2 h at room temperature. After thorough rinsing, the nuclei were stained with DAPI (94010, Vector Laboratories, Burlingame, CA, USA). All slices were photographed using a confocal fluorescence microscope (BX63, Olympus Optical Ltd, Tokyo, Japan). The number of immunoreactive cells in predefined areas were quantified using ImageJ software (Media Cybernetics Inc., Rockville, MD, USA). Six different fields for each mouse and six mice for each group were counted. All counts were conducted by blinded observers.

Western blotting assay

Western blotting was Carried out as previously described[11]. Cortical sections 1.0 to 2.0 mm from ipsilateral brain tissue was harvested and homogenized in cold RIPA buffer (C1053, Applygen, Beijing, China) plus protease inhibitor cocktail (G2006, Servicebio, Wuhan, China). The homogenates were centrifuged at 4 °C at 10, 000 × g for 30 min, and then the supernatants were harvested. Protein content was determined with the BCA kit (G2026, Servicebio, Wuhan, China). Protein samples (20 μl/lane) were separated by electrophoresis on 4 – 15% sodium dodecyl sulfate-polyacrylamide gels and then transferred onto PVDF membranes (Millipore, Billerica, MA, USA). Membranes were then put into 5% non-fat milk with PBS/0.1% Tween and blocked for 1 h, followed by incubation overnight with mouse anti-NLPR3 (1:1,000; ab4207, Cell Signaling Technology, Boston, USA), anti-NOX2 (1:1,000; ab18256, Abcam, Cambridge, England), anti-Beclin-1 (1:1,000; ab18256, Abcam, Cambridge, England), anti-IL-1β (1:1,000; ab12703, Cell Signaling Technology, Boston, USA), anti-COX-2 (1:1,000; 4842, Cell Signaling Technology, Boston, USA) and anti-LC3-B (1:1,000; 2775, Cell Signaling Technology, Boston, USA) at 4 °C. After washing with PBS/0.1% Tween, the membrane was incubated with IRDye-labeled secondary antibody (1:10,000; c60405-05, Li-Cor Bioscience, USA) at room temperature for 1–2 h. Images were acquired with the Odyssey Western Blot Analysis system (LI-COR, Lincoln, NE, USA). The relative band intensity was calculated using Quantity One v4.6.2 software (Bio-Rad Laboratories, Hercules, USA) and then normalized to the GADPH loading control. All above experiments were repeated three times.

Cytotoxicity Assay

MTT test was performed following the standard protocol[12] with a minor change. Cells were seeded in 96-well plates and incubated with different concentrations of **Mito-ONOO** (0, 2, 5, 10 and 20 μM, containing 1% DMSO in 100 μL DMEM). The cells were incubated in an atmosphere of 5/95 (*v*/*v*) of CO_2/air at 37 °C for 24 h. Next, 20 μL 5.0 mg/mL MTT solution was added into each well, followed by incubation for 4 h under the same condition. Then 150 μL DMSO was added into each well. After shaking for 10 min, the absorbance at 490 nm was measured by microplate reader (Synergy 2, BioTek Instruments Inc.). Cell survival rate was calculated by $A/A_0 \times 100\%$ (A and A_0 are the absorbance of the experimental group and control group, respectively).

General procedure for detection of H_2O_2, TNF-α and IL-1β concentration

BV-2 cells were cultured in 96-well plates and treated with NS-398、DPI、APO after bearing OGD/R process. After treatment, cell culture supernatants were collected for detection of TNF-α concentration using TNF-α ELISA kit (Invitrogen, ERA56RB) and IL-1β ELISA kit (Invitrogen, EHC002b) according to the manufacturer's instructions. Meanwhile, the cells were collected for measurement of cellular H_2O_2 concentration using the Amplex Red Hydrogen Peroxide Assay Kit (Invitrogen, A22188) according to recommended protocol as described earlier.

Histological staining of the tissue slices

After imaging, the mice were killed, and the brains and other tissues (heart, liver, spleen, lung, kidney, stomach) were collected for tissue analysis. Through a series of standard procedures, including fixation in 10% neutral buffered formalin, embedding into paraffin and sectioning at 3 μm thickness, the tissues were stained with hematoxylin-eosin (H&E). Thereafter, the prepared slices were examined by a digital microscope.

OGD/R model

OGD/R model of cells was performed by oxygen and glucose deprivation/reperfusion. BV-2 cells at 80% confluence were harvested by scraping and transferred to confocal dishes to grow[13]. When the cells are adherent, the culture medium is changed to sugar-free DMEM and cultured in a three-gas incubator for 12 hours without oxygen. Afterwards, these cells were incubated with high-glucose DMEM in a 5 % CO_2 and 95% O_2 atmosphere for different time. Then, the cells were incubated with **Mito-ONOO** for 30 min and washed with PBS for three times to perform two-photon confocal imaging.

Two-photon fluorescence cell imaging

Two days before imaging, the cells were passed and plated into glass-bottomed dishes (NEST). For labeling, the cells were washed with serum-free DMEM and then incubated with 5 μM **Mito-ONOO** (containing 1% DMSO in serum-free DMEM) for 30 min at 37 °C.

Calculation of Mean Fluorescence Intensity

The mean fluorescence density was measured by Image-Pro Plus (v. 6.0) and

calculated *via* the equation (mean density = $IOD_{sum}/area_{sum}$), where IOD and area were integral optical density and area of fluorescent region.

Measurement of ROS

To assess ROS production, the brain was carefully and quickly isolated and cut into 4.0 μm sections and placed on chilled microscope slides. The samples were incubated in physiological saline solution containing 10 μmol dihydroethidium (DHE; Sigma-Aldrich) for 30 min at 37 °C in the dark room. The brain was washed twice with PSS and placed under automatic fluorescence microscope (BX63, Olympus Optical Ltd, Tokyo, Japan).

Middle cerebral artery occlusion (MCAO) model

All animal procedures were performed in accordance with the Guidelines for Care and Use of Laboratory Animals of South-Central University for Nationalities and experiments were approved by the Animal Ethics Committee of College of Biology (South-Central University for Nationalities). Wild-type C57BL/6J mice (n = 300; 25–30 g) were purchased from Hubei Experimental Animal Research Center. (Hubei, China; No. 43004700018817, 43004700020932). All animal experimental protocols were approved by the Animal Experimentation Ethics Committee of South-Central University for Nationalities (No. 2020-scuec-043) and were conducted according to the Animal Care and Use Committee guidelines of South-Central University for Nationalities. Animals were housed in a room with controlled humidity (65 ± 5%) and temperature (25 ± 1 °C), under a 12/12-hour light/dark cycle with free access to food and water for at least 1 week before the experiments. MCAO was induced using a previously described method with slight modifications[14]. In brief, C57BL/6J wild-type mice were anesthetized with 5% isoflurane in O_2 by facemask, followed by ligation of the left middle cerebral artery with 6-0 monofilament (Doccol Corp., Redlands, CA, USA). After 1 h of occlusion, the monofilament was removed to initiate reperfusion. A homeothermic heating pad was employed to monitor and stabilize the mice body temperature at 37 ± 0.5 °C. The same procedure, but without monofilament ligation, was performed on sham-operated mice.

Measurement of infarct volume and neurological deficit

Wild-type C57BL/6J mice mentioned above were deeply anesthetized and euthanized with an overdose of isoflurane and decapitated 1 and 3 days after MCAO (i.e., after 3days of reperfusion). The brains were collected after transcranial perfusion

by saline followed with 4% paraformaldehyde. Brain tissues were cut into 1-mm coronal sections, and then dipped in 2% 2,3,5-triphenyltetrazolium chloride (TTC) (17779, Sigma-Aldrich, United States) for staining. The infarct volume was measured and analyzed by a blinded observer using ImageJ v1.37 (NIH, Bethesda, MA, United States), as described previously[15-17], then normalized and presented as a percentage of the non- ischemic hemisphere to correct for edema[18]. Neurological deficit scores were evaluated 3 days after MCAO as described previously[19]. The score ranged from 0 (without observable neurological deficit) to 4 (no spontaneous motor activity and loss of consciousness).

Assessment of neurological deficit

Neurological deficit scores were evaluated 3, 7 and 14 days after MCAO as described previously[15]. The score ranged from 0 (without observable neurological deficit) to 4 (no spontaneous motor activity and loss of consciousness).

Rotating beam walking test

The rotating beam walking test was used to evaluate neurological deficits in coordination and integration of movement in mice after MCAO[20]. The mice were trained to walk along a 100 cm rotating wood beam (80 mm in diameter, approximately 80 cm above the floor, at 3 rpm rotation) for 3 days (3 trials per day), then tested before and at 1 and 3 days after stroke. The walking time at each time point for each mouse was then recorded.

Rotarod test

Accelerating rotarod (SD Instruments, San Diego, CA) Instruments were used to test the motor coordination ability of the mice. Each MCAO model mouse was placed on a 2.75 cm diameter rotating rod every other day before MCAO onset for a total of 9 training days, and the rotation speed of rod increased from 5 to 10 rpm every 5 min. The time between the beginning of mouse staying on the rod and falling from the rod was determined up to a maximum duration of 300 seconds. After training period, MCAO surgery was conducted and Rotarod testing was performed by a blinded observer on 0, 1, 3 day post-stroke. The scores were calculated by averaging the three repetitive times records of each mouse each day.

In vivo imaging studies

After the MCAO model of Wild-type C57BL/6J mice mentioned above was

successfully established, **Mito-ONOO** (100 μL, 200 μM) was injected through the tail vein, and the mice were anesthetized with isoflurane before fluorescence imaging using a Bruker *in vivo* imaging system. Whereafter, the mice were anesthetized and dissected to remove the mouse brain tissue, and a 300 μm section was prepared with a microtome. Two-photon excited tissue fluorescence images were obtained by Zeiss LSM 710 multiphoton laser scanning confocal microscope.

Statistical analysis

Statistical Product and Service Solutions (SPSS) software 19.0 was used for the statistical analysis. The error bars shown in the figures represented the mean ± s.d. Differences were determined with a one-way analysis of variance (ANOVA) followed by LSD test. Statistical significance was assigned at $^*P < 0.05$, $^{**}P < 0.01$ and $^{***}P < 0.001$. Sample size was chosen empirically based on our previous experiences and pre-test results. No statistical method was used to predetermine sample size and no data were excluded. The numbers of animals or samples in every group were described in the corresponding figure legends. The distributions of the data were normal. All experiments were done with at least three biological replicates. Experimental groups were balanced in terms of animal age, sex and weight. Animals were all caged together and treated in the same way. Appropriate tests were chosen according to the data distribution. Variance was comparable between groups in experiments described throughout the manuscript.

2. Synthesis of Mito-ONOO and A-ONOO

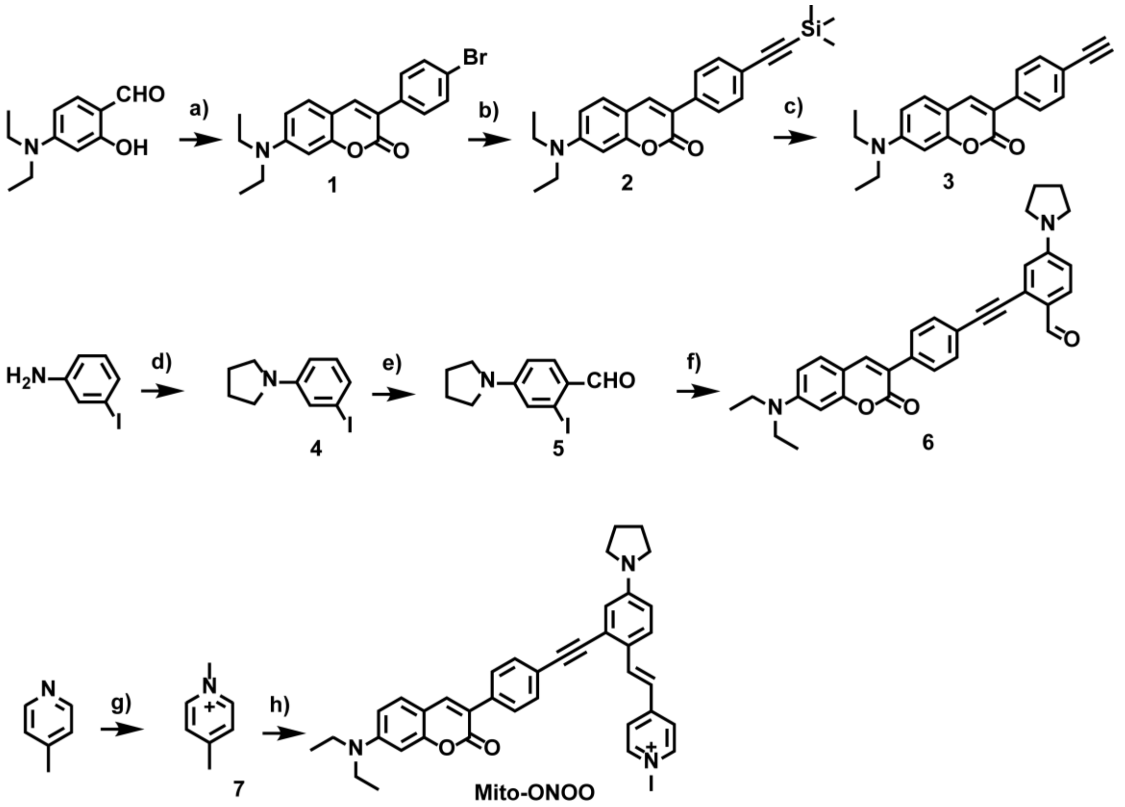

Scheme S1 Synthetic route of **Mito-ONOO**. Reagents and conditions: a) 4-bromophenylacetic acid, pyridine, acetic anhydride, reflux, 12 h. b) $Pd(PPh_3)_2Cl_2$, CuI, PPh_3, trimethylsilylacetylene, NEt_3, THF, Ar, 80 °C, 12 h. c) TBAF, THF, R.T., 1 h. d) 1,4-dibromobutane, K_2CO_3, anhydrous acetonitrile, reflux, 12 h. e) $POCl_3$, DMF, anhydrous 1,2-dichloroethane, 0 °C to reflux, 6 h. f) $Pd(PPh_3)_2Cl_2$, CuI, PPh_3, compound **3**, NEt_3, THF, Ar, 80 °C, 12 h. g) compound **4**, pyridine, anhydrous EtOH, Ar, 80 °C, 12 h. h) CH_3I, toluene, 110 °C, 6 h.

Synthesis of compound **1**. To 4-bromophenylacetic acid (4.8 g, 22.5 mmol) and 4-diethylamino-salicylaldehyde (4.3 g, 27.9 mmol) was added pyridine (2.25 mL, 28 mmol). The reaction mixture was then dissolved in acetic anhydride (5 mL) and refluxed for 12 h. The reaction, solvent was then removed and the crude residue was purified by column chromatography (1:6 v/v ethyl acetate/petroleum ether) to give the product as yellow solid. Yield: 5.7 g (68.7%). ^{1}H NMR (400 MHz, $CDCl_3$) δ 7.72 (s, 1H), 7.70 – 7.63 (m, 2H), 7.55 – 7.45 (m, 2H), 7.32 (d, J = 8.8 Hz, 1H), 6.62 (dd, J = 8.8, 2.1 Hz, 1H), 6.55 (d, J = 2.2 Hz, 1H), 3.43 (q, J = 7.1 Hz, 4H), 1.23 (t, J = 7.1 Hz, 6H).

Synthesis of compound **2**. To compound **1** (3.3 g, 8.9 mmol) was added $Pd(PPh_3)_2Cl_2$ (630 mg, 0.89 mmol), CuI (170 mg, 0.89 mmol), PPh_3 (480 mg, 1.8 mmol) and trimethylsilylacetylene (3.78 mL, 26.7 mmol). NEt_3 (5 mL) and THF (25 mL) were then added and the reaction mixture was refluxed for 12 h under an inert Ar atmosphere. The solvent was then removed and the crude residue was purified by column chromatography (1:6 v/v ethyl acetate/petroleum ether) to give the product as yellow solid. Yield: 3.0 g (88.7%). ^{1}H NMR (400 MHz, $CDCl_3$) δ 7.72 (s, 1H), 7.70 – 7.63 (m, 2H), 7.55 – 7.45 (m, 2H), 7.32 (d, *J* = 8.8 Hz, 1H), 6.62 (dd, *J* = 8.8, 2.1 Hz, 1H), 6.55 (d, *J* = 2.2 Hz, 1H), 3.43 (q, *J* = 7.1 Hz, 4H), 1.23 (t, *J* = 7.1 Hz, 6H), 0.31 – 0.20 (m, 9H).

Synthesis of compound **3**. Compound **2** (2.65 g, 6.81 mmol) was dissolved in THF (100 mL) and 1 M TBAF solution in THF (6.81 mL, 6.81 mmol) was added. The reaction was stirred at R.T. for 1 h. The solvent was then removed and the crude residue was purified by column chromatography (1:3 v/v ethyl acetate/petroleum ether) to give the product as yellow solid. Yield: 1.74 g (80.6%). ^{1}H NMR (400 MHz, $CDCl_3$) δ 7.70 (dd, *J* = 9.3, 7.6 Hz, 3H), 7.53 (d, *J* = 8.4 Hz, 2H), 7.32 (d, *J* = 8.8 Hz, 1H), 6.62 (dd, *J* = 8.8, 2.1 Hz, 1H), 6.54 (d, *J* = 2.2 Hz, 1H), 3.44 (q, *J* = 7.1 Hz, 4H), 3.12 (s, 1H), 1.23 (t, *J* = 7.1 Hz, 6H).

Synthesis of compound **4**. 3-iodoaniline (5.0 g, 22.8 mmol) and K_2CO_3 (9.5 g, 68.4 mmol) were dissolved in anhydrous acetonitrile (100 mL) and 1,4-dibromobutane (5.9 g, 27.4 mmol) was added. The reaction was reflux for 12 h. The solvent was then removed and the crude residue was purified by column chromatography (1:10 v/v dichloromethane/petroleum ether) to give the product as yellow oil. Yield: 5.43 g (87%). ^{1}H NMR (400 MHz, $CDCl_3$) δ 7.02 – 6.86 (m, 3H), 6.56 – 6.48 (m, 1H), 3.31 – 3.21 (m, 4H), 2.04 – 1.96 (m, 4H).

Synthesis of compound **5**. $POCl_3$ (11 mL) and DMF (10 mL) were stirred together in a flask for 30 min at 0 °C before a solution of compound **4** (5.0 g, 18.3 mmol) in anhydrous 1,2-dichloroethane (40 mL) was added slowly. The resulting mixture was reflux for 6 h before being poured into ice water. Brownish yellow precipitates were collected via suction filtration and washed with water. The solid was dissolved in CH_2Cl_2 and residual H_2O was removed with anhydrous $MgSO_4$. Upon suction filtration to remove solids, the filtrate was passed through a short column chromatography (1:4 v/v

dichloromethane/petroleum ether) to remove the colored impurities. Evaporation under reduced pressure afforded the product as a yellow crystalline solid. Yield: 4.8 g (87.3%). ^{1}H NMR (400 MHz, $CDCl_3$) δ 9.77 (s, 1H), 7.75 (d, *J* = 8.8 Hz, 1H), 7.00 (d, *J* = 2.4 Hz, 1H), 6.55 (dd, *J* = 8.8, 1.8 Hz, 1H), 3.48 – 3.24 (m, 4H), 2.12 – 1.98 (m, 4H).

Synthesis of compound **6**. To compound **5** (3.3 g, 8.9 mmol) was added $Pd(PPh_3)_2Cl_2$ (630 mg, 0.89 mmol), CuI (170 mg, 0.89 mmol), PPh_3 (480 mg, 1.8 mmol) and compound **3** (2.8 g, 8.9 mmol). NEt_3 (5 mL) and THF (25 mL) were then added and the reaction mixture was was stirred at R.T. for 6 h under an inert Ar atmosphere. The solvent was then removed and the crude residue was purified by column chromatography (1:6 v/v ethyl acetate/petroleum ether) to give the product as yellow solid. Yield: 2.0 g (47.2%). ^{1}H NMR (400 MHz, $CDCl_3$) δ 10.38 (s, 1H), 7.86 (d, *J* = 8.8 Hz, 1H), 7.75 (d, *J* = 6.5 Hz, 3H), 7.60 (d, *J* = 8.3 Hz, 2H), 7.34 (d, *J* = 8.8 Hz, 1H), 6.69 (s, 1H), 6.62 (d, *J* = 7.5 Hz, 1H), 6.56 (d, *J* = 13.0 Hz, 2H), 3.44 (dd, *J* = 13.1, 6.1 Hz, 8H), 2.07 (s, 4H), 1.42 (dd, *J* = 12.5, 6.4 Hz, 6H). HRMS (MALDI-DHB): calcd for $C_{32}H_{30}N_2O_3$ $[M + H]^+$ 491.2256 found 491.2308.

Synthesis of compound **7**. 4-Picoline (2.9 g, 31.4 mmol) was dissolved in dry toluene. Then methyliodide (5.3 g, 33.0 mmol) was added dropwise and refluxed for 6 h. The reaction was was cooled to room temperature and the pale pink precipitate was was filtered, washed with toluene five times and dried in a desiccator to give the product as white solid. Yield: 5.9 g (79.8%). ^{1}H NMR (400 MHz, d_6-DMSO) δ 8.81 (d, *J* = 6.5 Hz, 2H), 7.95 (d, *J* = 6.3 Hz, 2H), 4.26 (s, 3H), 2.58 (s, 3H).

Synthesis of **Mito-ONOO**. Compound **6** (200 mg, 0.41 mmol), Compound **7** (188 mg, 0.8 mmol) and piperidine (catalyst, 10 drops) were dissolved in absolute ethanol (20 mL). The mixture was refluxed for 9 h under an inert Ar atmosphere. After cooling to room temperature, the red solid was filtered. Then, the crude solid was purified by column chromatography (1:20 v/v methanol/dichloromethane) to give the product as red solid. Yield: 108.4 mg (45.6%). ^{1}H NMR (400 MHz, $CDCl_3$) δ 8.75 (d, *J* = 6.5 Hz, 2H), 8.11 (d, *J* = 16.1 Hz, 1H), 7.89 (s, 1H), 7.86 – 7.80 (m, 2H), 7.75 (d, *J* = 6.5 Hz, 2H), 7.67 (d, *J* = 9.0 Hz, 1H), 7.61 (d, *J* = 8.2 Hz, 2H), 7.41 (d, *J* = 8.9 Hz, 1H), 6.98 (d, *J* =

16.1 Hz, 1H), 6.69 (s, 1H), 6.62 (d, J = 8.7 Hz, 1H), 6.57 (d, J = 9.1 Hz, 1H), 6.53 (s, 1H), 4.39 (s, 3H), 3.44 (dd, J = 14.6, 7.5 Hz, 4H), 3.40 (d, J = 9.2 Hz, 4H), 2.15 – 1.99 (m, 4H), 1.23 (s, 6H). ^{13}C NMR (100 MHz, $CDCl_3$) δ 186.63, 152.77, 152.04, 143.58, 133.87, 133.81, 123.11, 121.45, 117.33, 111.54, 106.37, 103.83, 83.98, 48.82, 48.36, 42.31, 37.85, 28.41, 20.14, 13.12, 10.43. HRMS (MALDI-DHB): calcd for $C_{44}H_{44}N_3O_2$ $[M]^+$ 580.2959 found 580.2923.

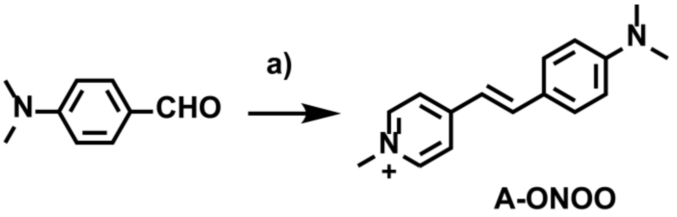

Scheme S2. Synthetic route of **A-ONOO**. *Reagents and conditions:* a) Compound **7**, pyridine, anhydrous EtOH, Ar, 80 °C, 12 h.

Synthesis of **A-ONOO**.

4-(dimethylamino) benzaldehyde (200 mg, 1.34 mmol), Compound **7** (348 mg, 2 mmol) and piperidine (catalyst, 10 drops) were dissolved in absolute ethanol (20 mL). The mixture was refluxed for 9 h under an inert Ar atmosphere. After cooling to room temperature, the red solid was filtered. Then, the crude solid was then purified by column chromatography (1:20 v/v methanol/dichloromethane) to give the product as red solid. Yield: 316.4 mg (64.5%). ^{1}H NMR (400 MHz, d_6-DMSO) δ 8.70 (d, J = 6.9 Hz, 2H), 8.06 (d, J = 6.9 Hz, 2H), 7.92 (d, J = 16.1 Hz, 1H), 7.60 (d, J = 8.9 Hz, 2H), 7.18 (d, J = 16.1 Hz, 1H), 6.79 (d, J = 9.0 Hz, 2H), 4.18 (s, 3H), 3.02 (s, 6H).

3. Supplementary Figures

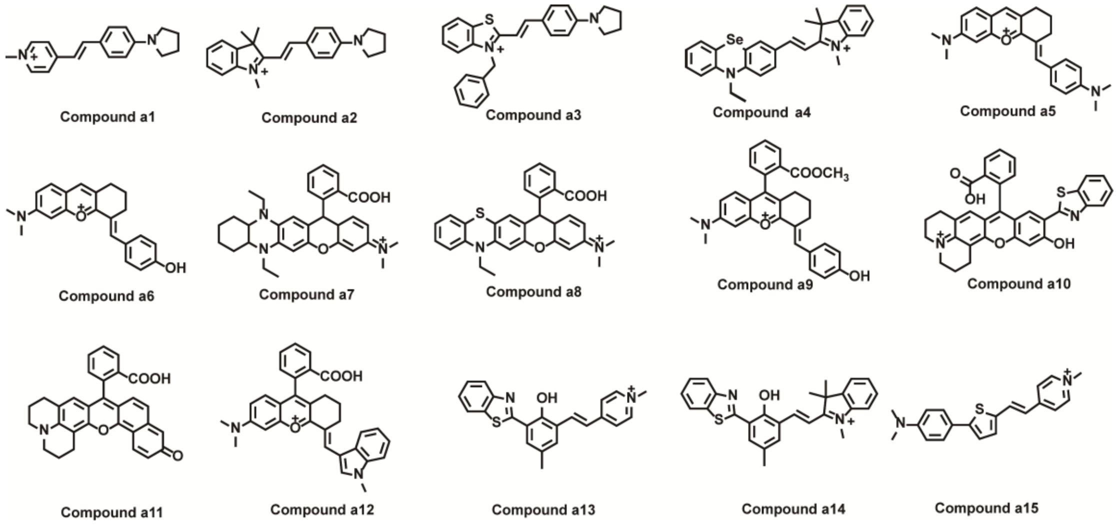

Fig. S1. Structure of compounds **a1**-**a15**.

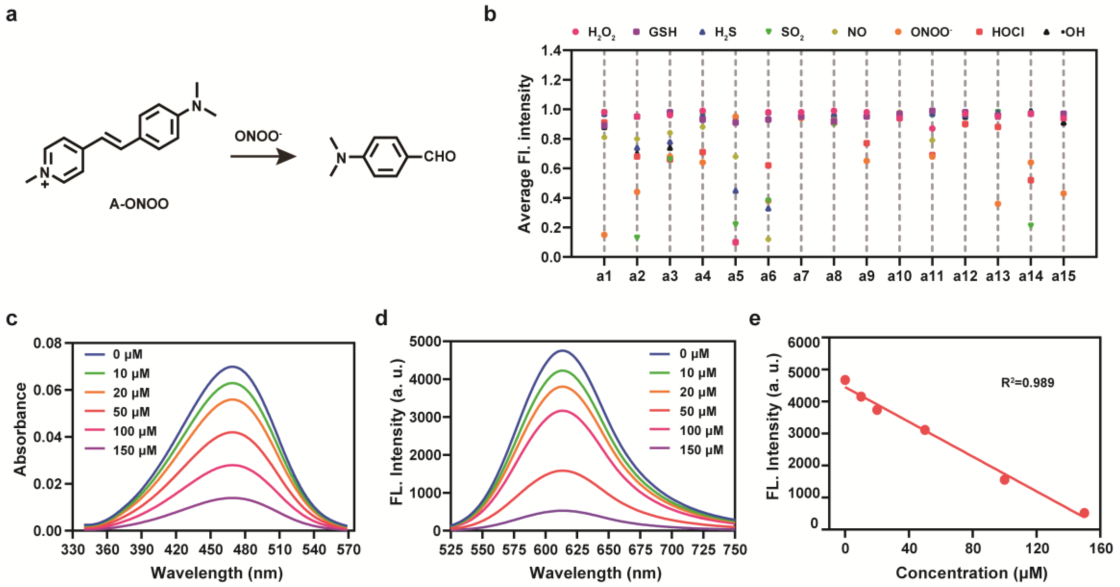

Fig. S2. a) Structure of **A-ONOO** probe and its sensing mechanism towards $ONOO^-$; b) Fluorescence response of compounds **a1**−**a15** (5 μM, structure of compounds **a1**−**a15** are shown in Fig. S1) to various agents in an aqueous solution (PBS, 10 mM, pH 7.4, 0.9% NaCl, containing 40% EtOH) (100 μM for H_2O_2: Red; 100 μM for GSH: Olive; 100 μM for H_2S: Blue; 150 μM for SO_2: Green; 150 μM for NO: Wine; 25 μM for $ONOO^-$: Orange; 25 μM for HClO: Red; 25 μM for·OH: Black). The test solution was kept at 37 °C for 20 min before the data was recorded; Absorption c) and fluorescence d) spectra of **A-ONOO** (10 μM) in presence of increasing SIN-1 concentration (0, 10, 20, 50, 100 and 150 μM) λ_{ex} = 476 nm; e) Calibration curve of fluorescence intensity for the determination of $ONOO^-$.

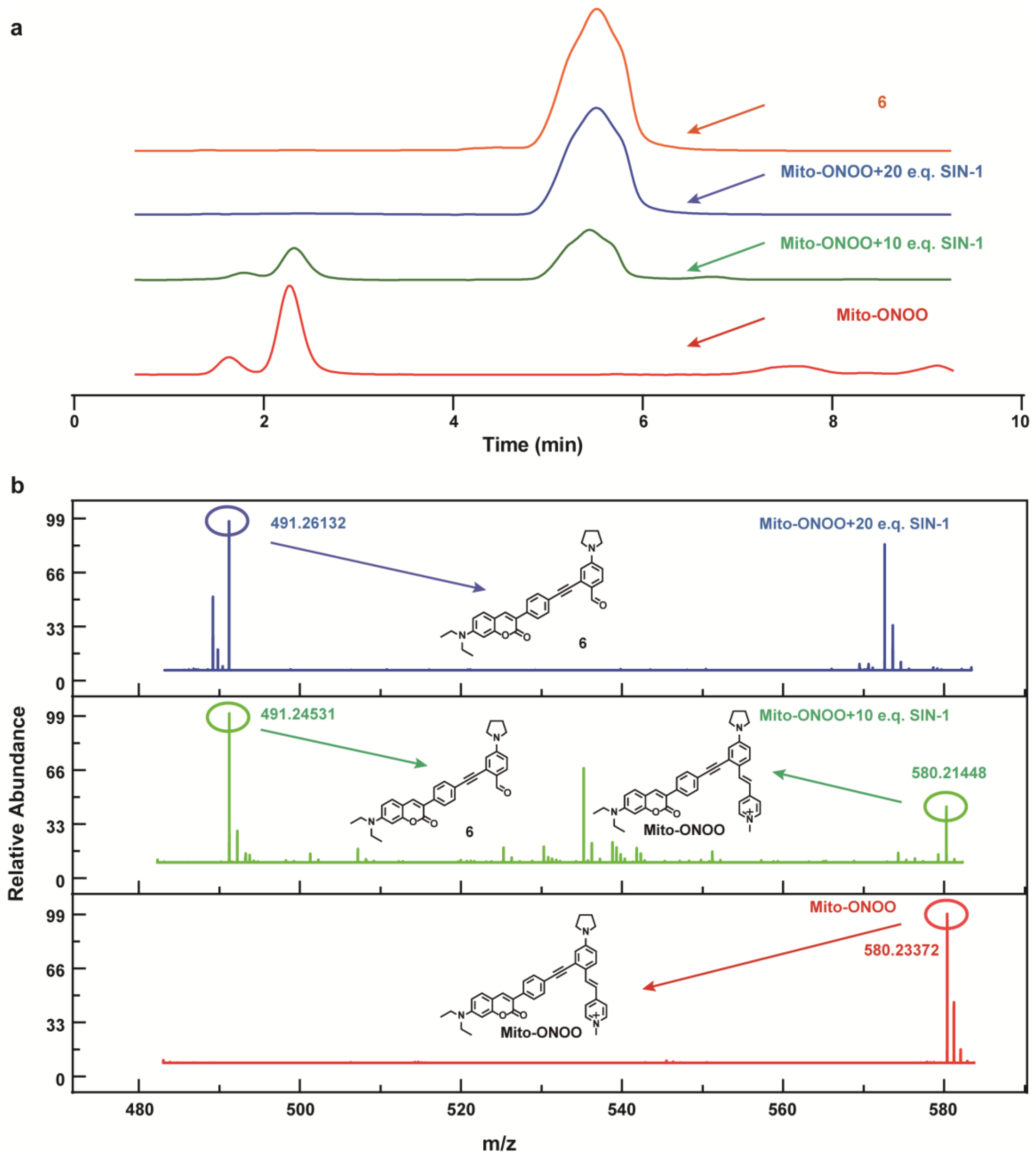

Fig. S3. (a) HPLC traces of the samples: **Mito-ONOO** (5 μM, Orange line), **Mito-ONOO** (5 μM) after reacting with 10 e.q. SIN-1 in PBS buffer for 2 min (blue line), **Mito-ONOO** (5 μM) after reacting with 20 e.q. SIN-1 in PBS buffer for 2 min (green line) and **6** (5 μM, red line). (b) HRMS traces of **Mito-ONOO** upon addition of top: SIN-1 (20 e.q.), middle: SIN-1 (10 e.q.) and bottom: SIN-1 (0 e.q.).

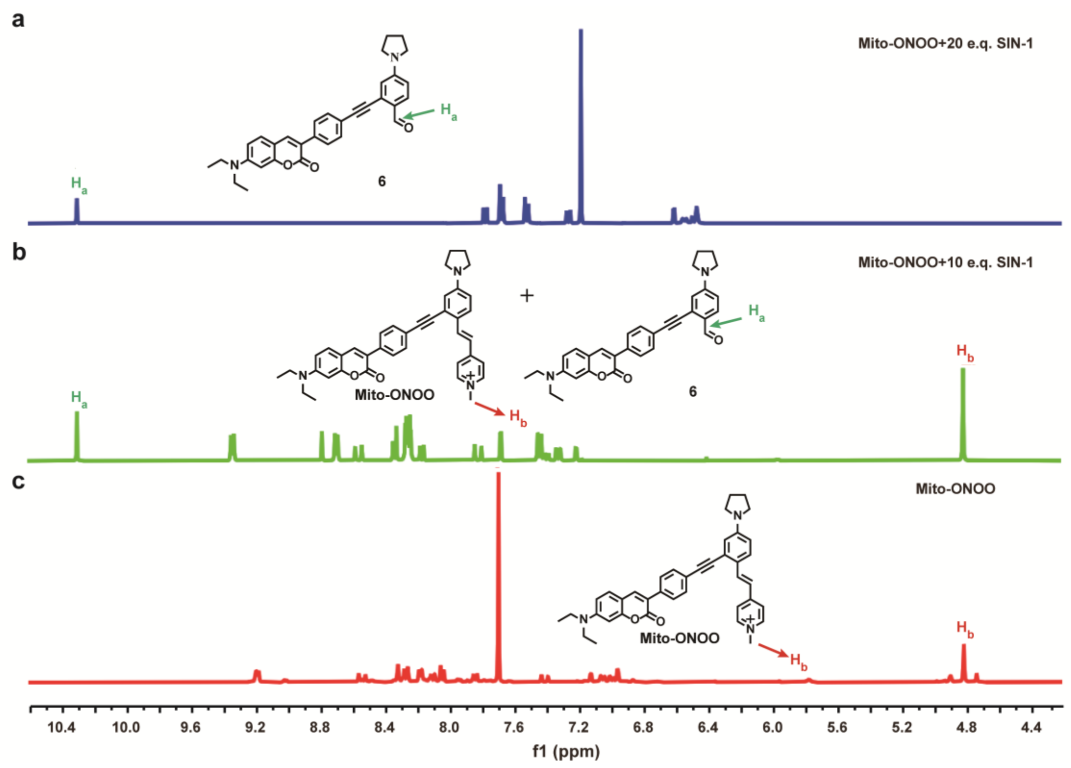

Fig. S4. ^{1}H NMR spectra of **Mito-ONOO** upon addition of a) SIN-1 (20 e.q.), b) SIN-1 (10 e.q.) and c) SIN-1 (0 e.q.) in $CDCl_3$.

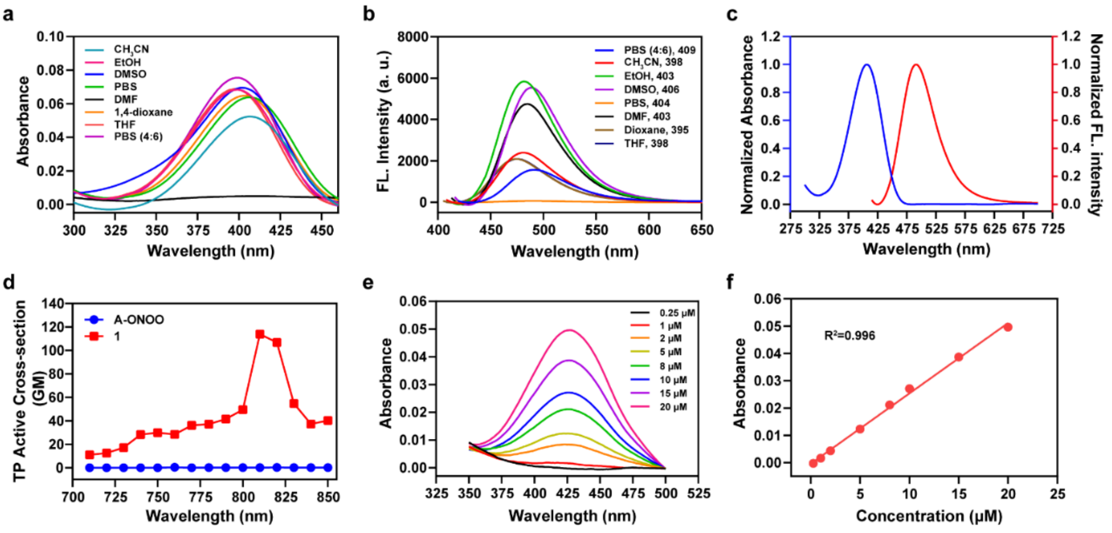

Fig. S5. a) Absorption spectra (10 μM) and b) Fluorescence emission spectra of **1** in different solvents (λ_{ex} = 416 nm). c) Normalized absorption (blue line) and fluorescence (red line) of **1** in PBS (10 mM, pH 7.4, 0.9% NaCl, containing 40% EtOH). d) Two-photon active cross section (Φδ) of **A-ONOO** (blue line) and **1** (red line) λ_{ex} = 780-850 nm. e) absorption spectra and f) plot of absorption against the concentration of **Mito-ONOO** in water (containing 1% EtOH).

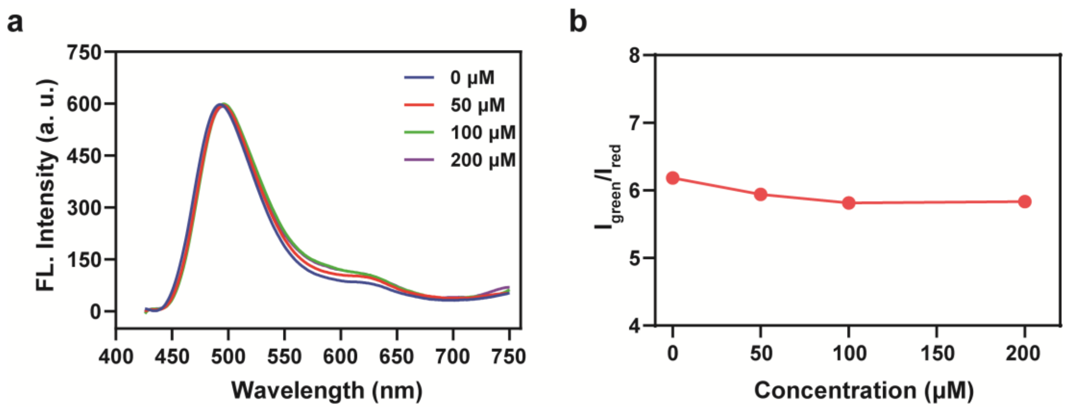

Fig. S6. a) Fluorescence spectra of **Mito-ONOO** (10 μM) in presence of increasing SIN-1 concentration (0, 50, 100, 200 μM) λ_{ex} = 416 nm; b) Calibration curve for the determination of $ONOO^{-}$.

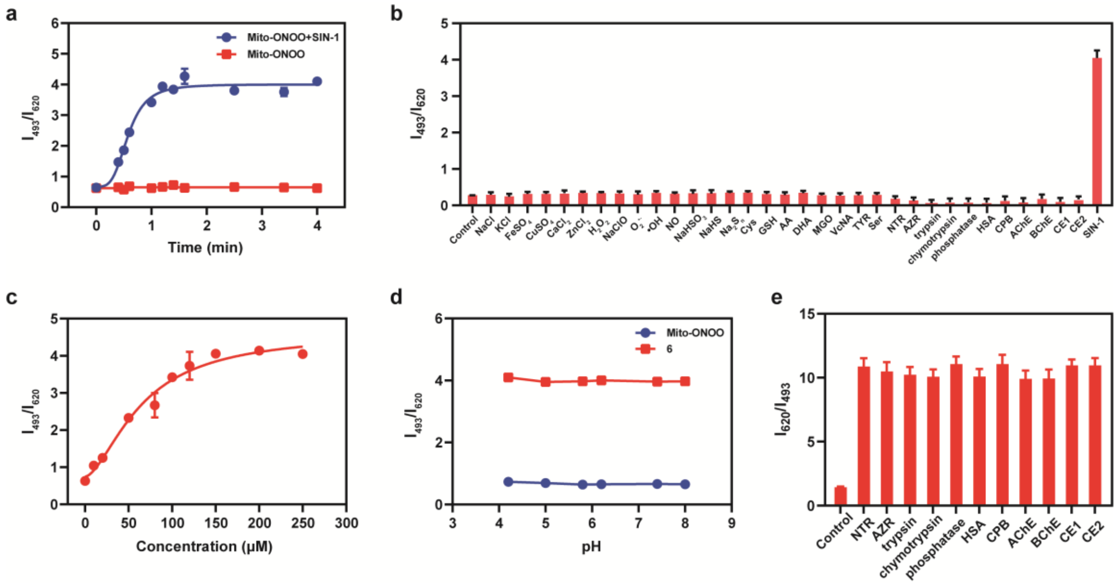

Fig. S7. a) Plot of fluorescence intensity of **Mito-ONOO** (10 μM) *vs* the reaction time in the absence and presence of SIN-1, (from bottom to top): 0 μM (control), 150 μM; b) Fluorescence response of **Mito-ONOO** (10 μM) to 200 μM various species: control, NaCl, KCl, $FeSO_4$, $CuSO_4$, $CaCl_2$, $ZnCl_2$, H_2O_2, NaClO, $O_2^{\bullet-}$, •OH, NO, $NaHSO_3$, NaHS, Na_2S_n, Cys, GSH, AA, DHA, MGO, VcNA, TYR, Ser, NTR, AZR, NADH, NADPH, trypsin, chymotrypsin, phosphatase, HSA, CPB, AChe, BChe, CH1, CE2 and SIN-1. c) Calibration curve for the determination of increasing SIN-1 concentration (0, 2, 5, 15, 30, 50, 100, 120, 150, 200 and 250 μM). d) Fluorescence intensity ratio of **Mito-ONOO** in absence (blue line) and presence (red line) of SIN-1 in PBS (10 mM, pH 4.4-8.0). e) Fluorescence response of **Mito-ONOO** (10 μM) to various species: control, NTR, AZR, NADH, NADPH, trypsin, chymotrypsin, phosphatase, HSA, CPB, AChe, BChe, CH1 and CE2.The measurements of a-d using **Mito-ONOO** were evaluated in PBS buffer (10 mM, pH 7.4, containing 30% EtOH and 40% glycerol). The measurements of e using **Mito-ONOO** were evaluated in PBS buffer (10 mM, pH 7.4, containing 30% EtOH). λ_{ex} = 416 nm. All of the data represent the mean of three replicates and the error bars indicate the SD.

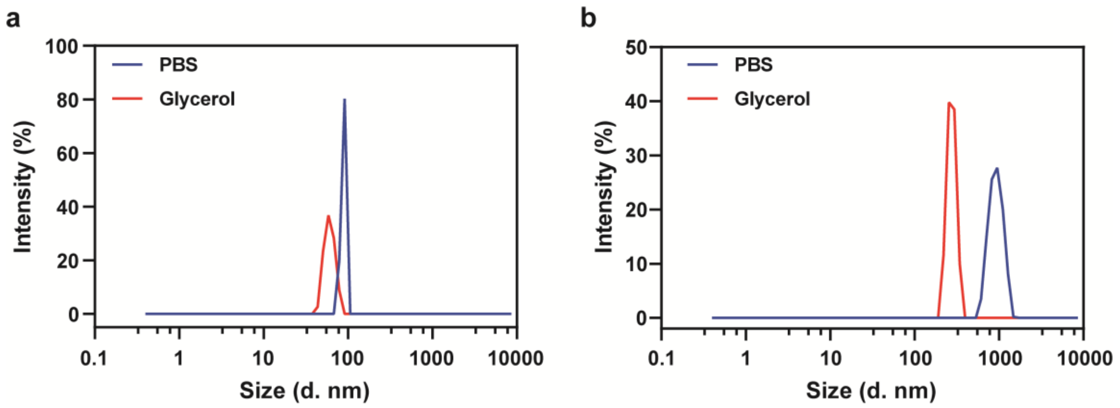

Fig. S8. Dynamic light scattering results for a) **Mito-ONOO** in absence (blue line) and presence (red line) of glycerol in PBS buffer; b) **6** in absence (blue line) and presence (red line) of glycerol in PBS buffer.

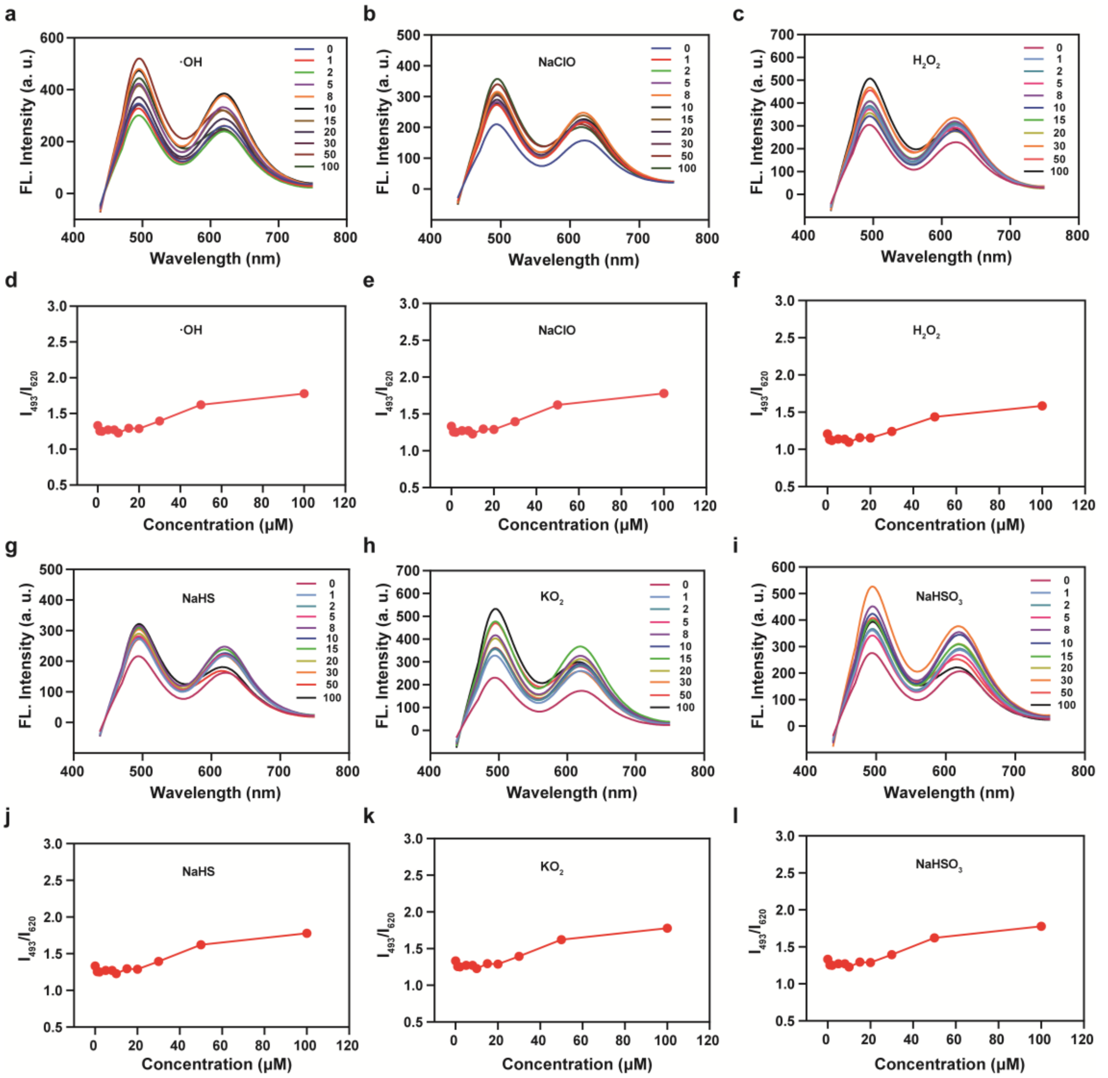

Fig. S9. Fluorescence spectra of **Mito-ONOO** (10 μM) treated with various agents (0-100 μM) in pH 7.4 PBS/EtOH (6/4). HClO; H_2O_2; •OH; KO_2; SO_3^{2-} and H_2S. Data were recorded 20 min after the addition of analytes at 37 °C λ_{ex} = 416 nm.

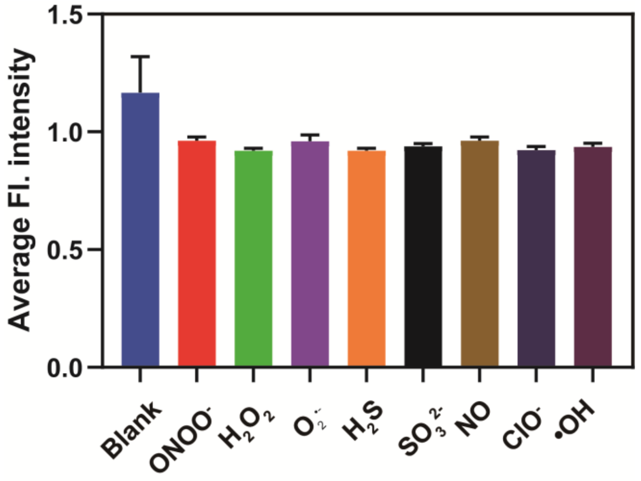

Fig. S10. Fluorescence intensities of **6** (10 μM, structure is shown in Scheme 1) to various agents in an aqueous solution (PBS, 10 mM, pH 7.4, 0.9% NaCl, containing 40% EtOH). λ_{ex} = 416 nm. Data represent the mean of three replicates and the error bars indicate the SD.

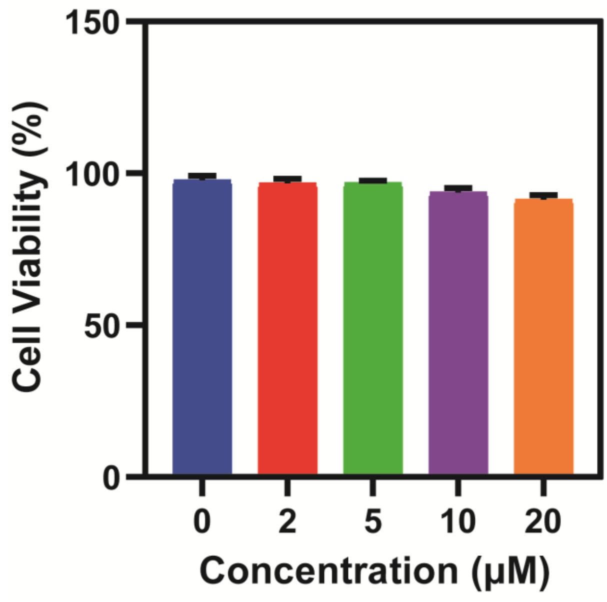

Fig. S11. MTT assay of BV-2 cells treated with different concentrations of **Mito-ONOO** (0, 2, 5, 10, 20 μM). Data represent the mean of three replicates and the error bars indicate the SD.

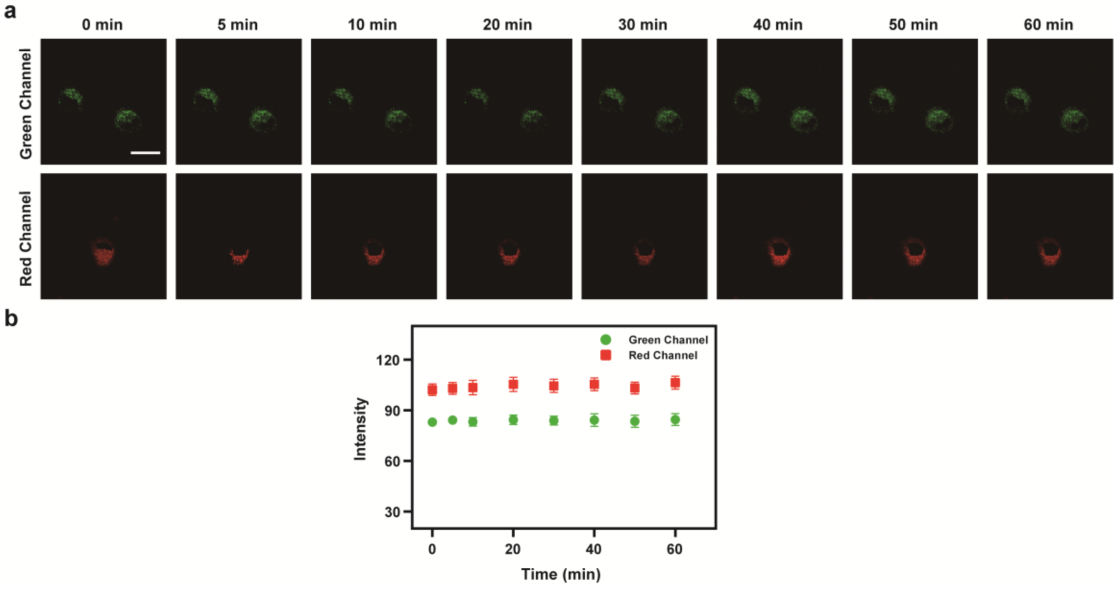

Fig. S12. a) Two-photon fluorescence imaging and b) time course of fluorescence changes of BV2 cells. λ_{ex} = 810 nm; λ_{em} = 450-530 nm (the green channel) and 600-700 nm (red channel). In b, the error bars indicate the SD. Scale bar: 50 μm.

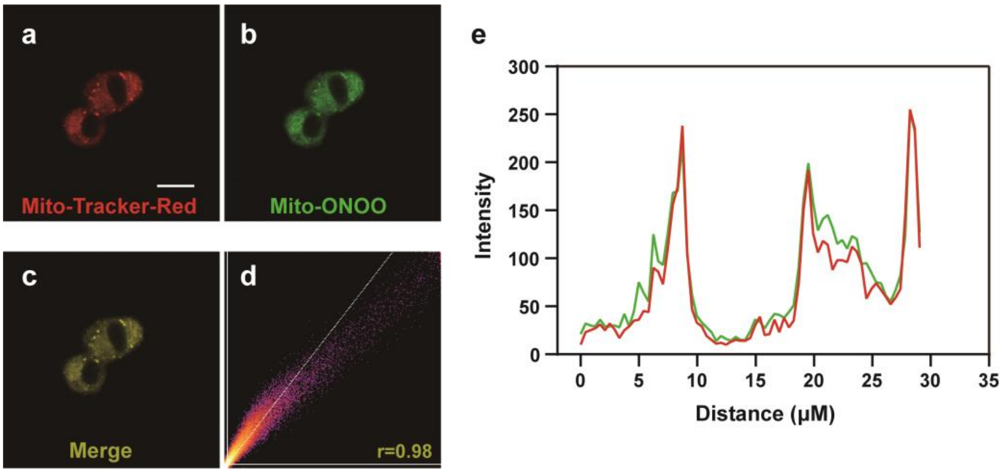

Fig. S13. Fluorescence images of BV-2 cells. Cells incubated with 10 μM probe **Mito-ONOO** and 100 nM **Mito-Tracker-Red** at 37 °C for 20 min in DMEM media supplemented with 10% FBS: a) **Mito-Tracker-Red** λ_{ex} = 560 nm and a scan range of 595–650 nm; b) probe **Mito-ONOO** in the presence of monensin λ_{ex} = 810 nm and a scan range of 450–530 nm; c) overlay of a) and b); d) Co-localization coefficient (Pearson's coefficient) of **Mito-ONOO** and **Mito-Tracker-Red** is 0.98. (e) Fluorescence intensity profile of the region of interest across cells in the red and green channels. Scale bar: 50 μm.

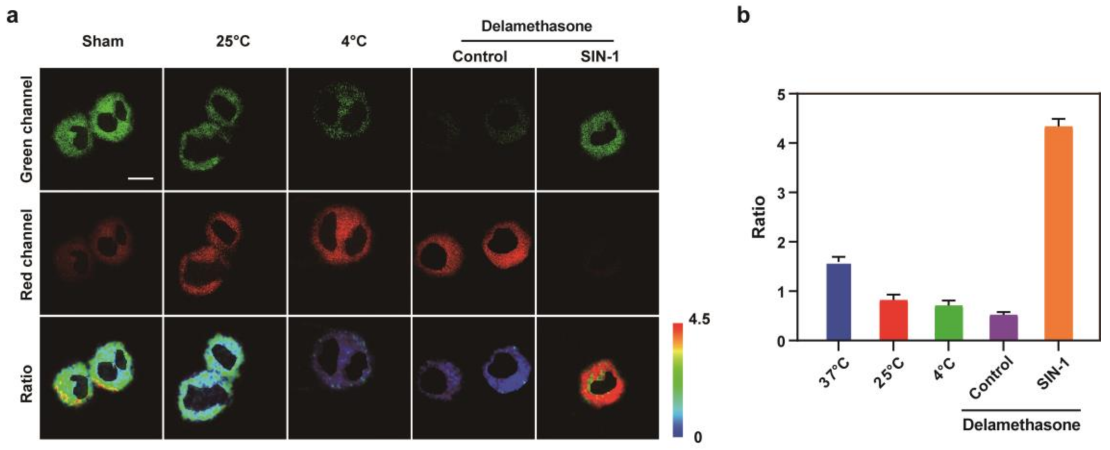

Fig. S14. Two-photon fluorescence images of BV-2 cells. a) Cells were incubated with **Mito-ONOO** (10 μM) only at 37 °C (n = 40 cells from three cultures), 25 °C (n = 32 cells from three cultures) and 4 °C (n = 28 cells from three cultures) for 30 min, respectively. Cells incubated with dexamethasone (5 mM, n = 44 cells from three cultures) at 37 °C for 30 min and then treated with SIN-1 for another 10 min. b) Mean intensity ratio (F_{green}/F_{red}) of a). Emissions were collected at the green channel (450-530 nm) and red channel (600-700 nm) λ_{ex} = 810 nm. Scale bar: 50 μm. Data represent the mean of three replicates and the error bars indicate the SD.

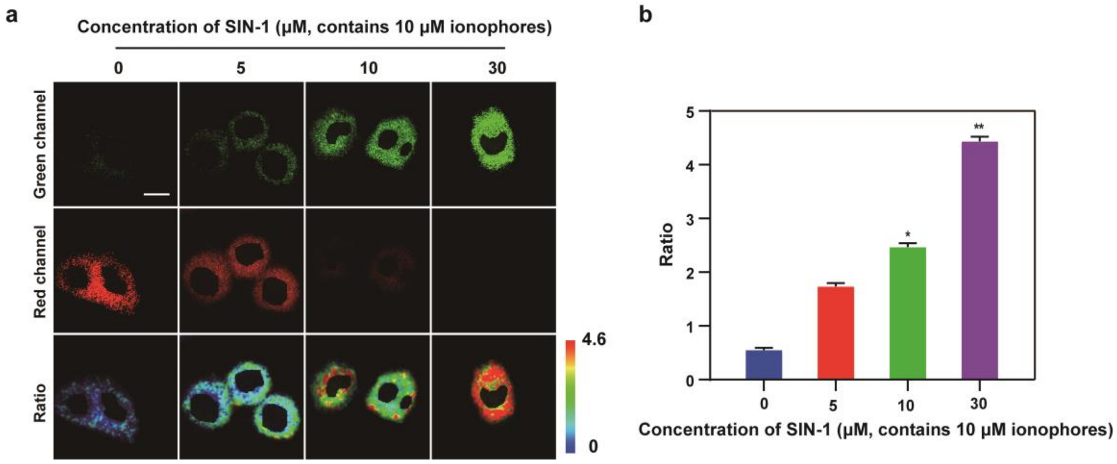

Fig. S15. a) Two-photon fluorescence images of BV-2 cells incubated with **Mito-ONOO** (10 μM), obtained under different concentrations of SIN-1 (0, 5, 10, 30 μM). b) Mean intensity ratio (F_{green}/F_{red}) of a). Emissions were collected at the green channel (450-530 nm) and red channel (600-700 nm) λ_{ex} = 810 nm. Scale bar: 50 μm. Data represent the mean of three replicates and the error bars indicate the SD (n = 18 cells from three independent cultures for each group). For all experiments in the presence of ionophores (monensin and nystatin) (10 μM).

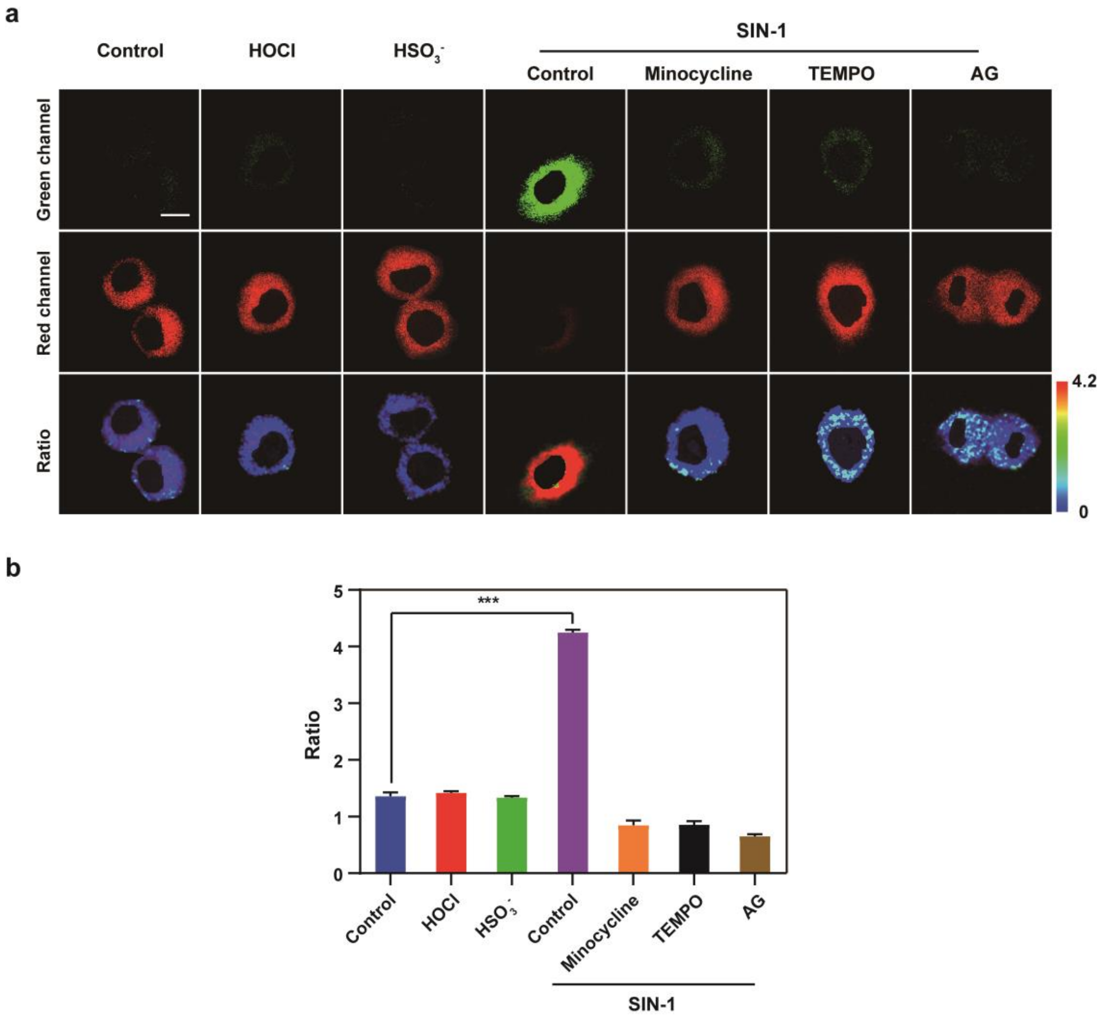

Fig. S16. a) Two-photon fluorescence images of BV-2 cells incubated with **Mito-ONOO** (10 μM) and treated with 1) control (n = 48 cells from three cultures); 2) HOCl (n = 37 cells from three cultures); 3) HSO_3^- (n = 40 cells from three cultures); 4) SIN-1 (n = 29 cells from three cultures); 5) SIN-1 and minocycline (n = 43 cells from three cultures); 6) SIN-1 and TEMPO (n = 38 cells from three cultures) and 7) SIN-1 and AG (n = 41 cells from three cultures); b) Mean intensity ratio (F_{green}/F_{red}) of a). Emissions were collected at the green channel (450-530 nm) and red channel (600-700 nm) λ_{ex} = 810 nm. Scale bar: 50 μm. Data represent the mean of three replicates and the error bars indicate the SD. Difference was analyzed by one-way ANOVA and Bonferroni post-test. ***$P < 0.001$. For all experiments in the presence of ionophores (monensin and nystatin) (10 μM).

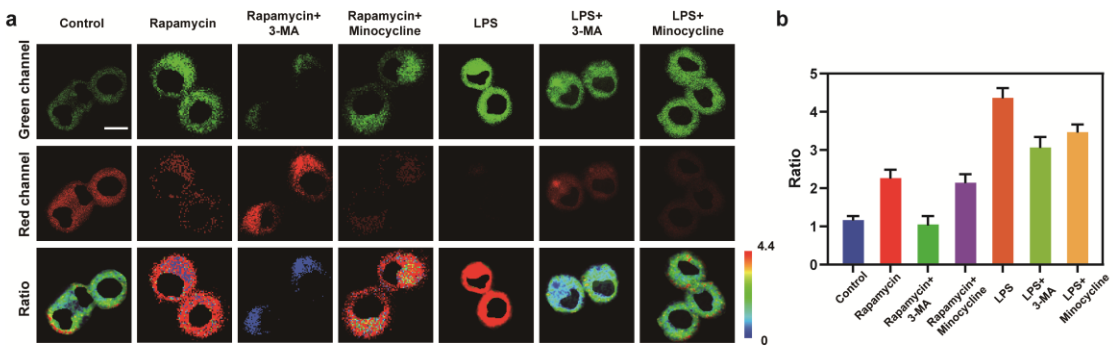

Fig. S17. a) Two-photon fluorescence images of BV-2 cells incubated with **Mito-ONOO** (10 μM) and treated with different treatment (Control, n = 47 cells from three cultures; Rapamycin alone, n = 42 cells from three cultures; Rapamycin in the presence of 3-MA, n = 39 cells from three cultures; and Rapamycin in the presence of minocycline, n = 38 cells from three cultures); LPS alone, n = 33 cells from three cultures; LPS in the presence of 3-MA, n = 43 cells from three cultures; and LPS in the presence of minocycline, n = 31 cells from three cultures). b) Mean intensity ratio (F_{green}/F_{red}) of a). Emissions were collected at the green channel (450-530 nm) and red channel (600-700 nm) λ_{ex} = 810 nm. Scale bar: 50 μm. Data represent the mean of three replicates and the error bars indicate the SD.

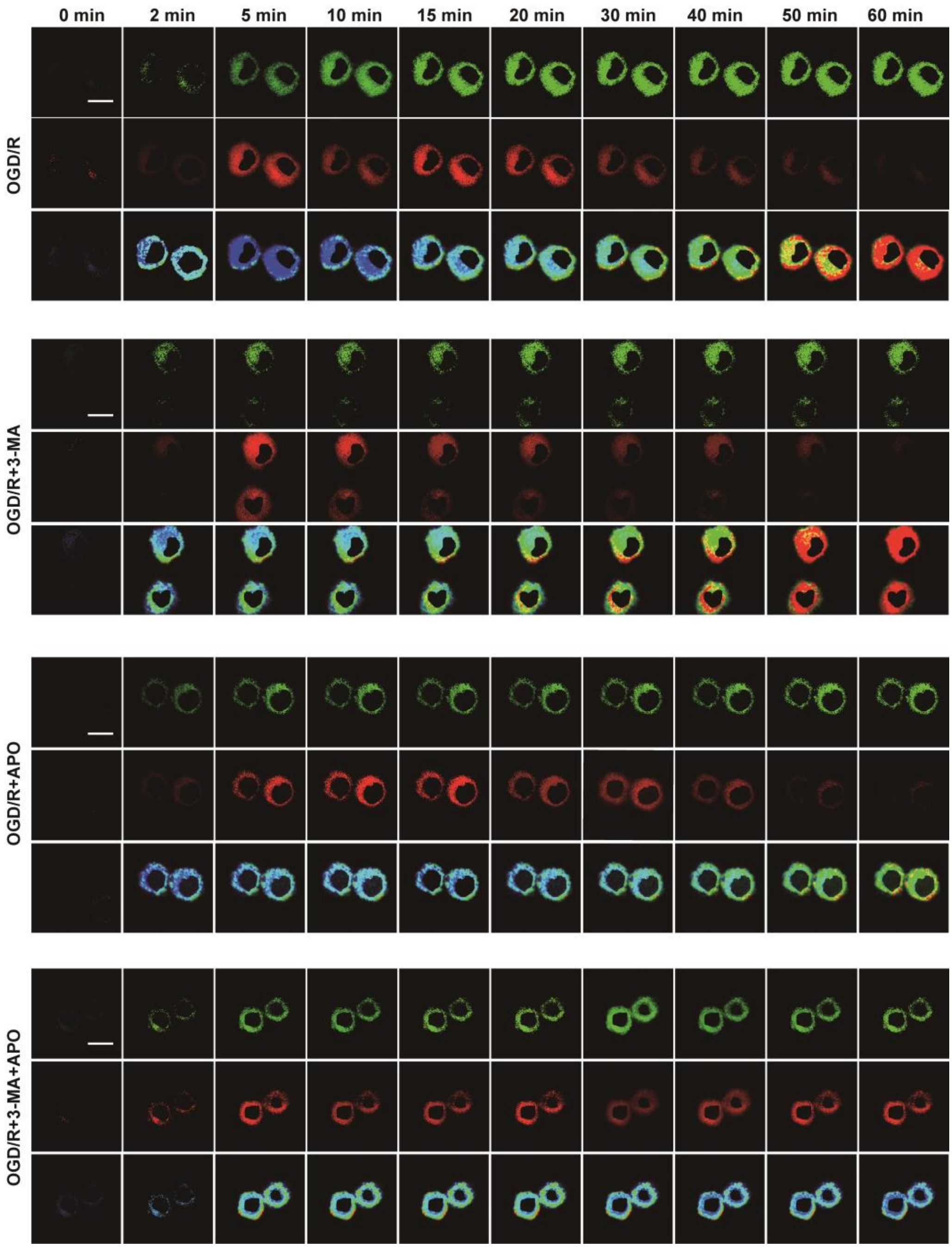

Fig. S18. Real-time two-photon fluorescence imaging of BV-2 cells incubated with **Mito-ONOO** (10 μM) during OGD/R with OGD/R (n = 43 cells from three cultures), OGD/R+3-MA (n = 33 cells from three cultures), OGD/R+APO (n = 41 cells from three cultures) and OGD/R+3-MA+APO (n = 38 cells from three cultures). Emissions were collected at the green channel (450-530 nm) and red channel (600-700 nm) λ_{ex} = 810 nm. Scale bar: 50 μm.

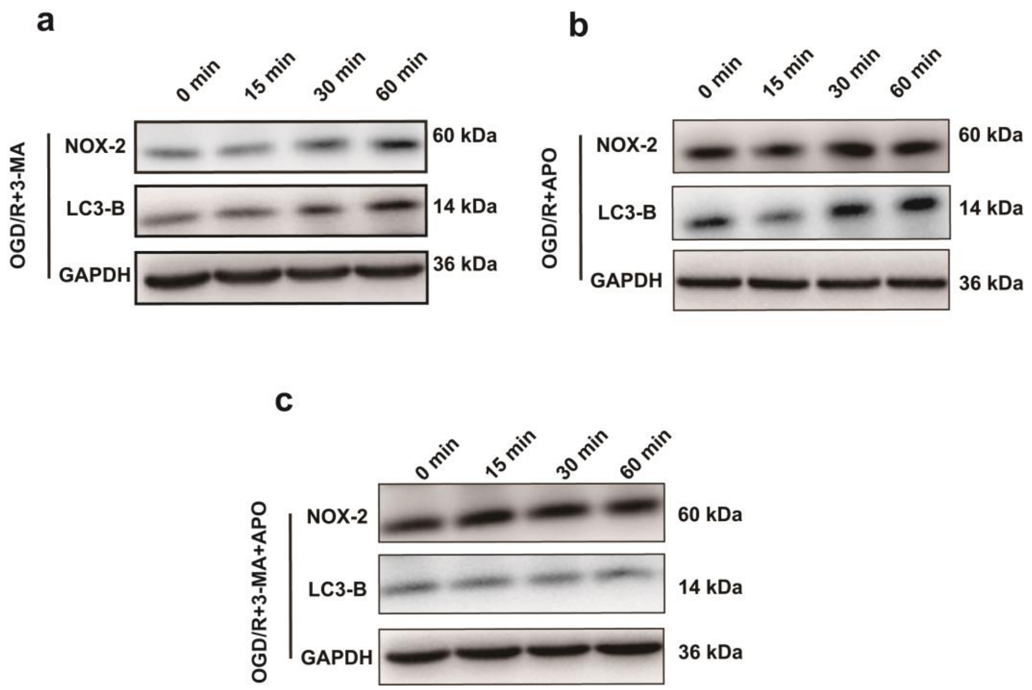

Fig. S19. Western blotting indicating the expression of NOX-2, LC3-II and GAPDH in BV-2 cells during OGD/R and pre-treated with 3-MA a), APO b) and 3-MA+APO c) at different times (0, 15, 30 and 60 min).

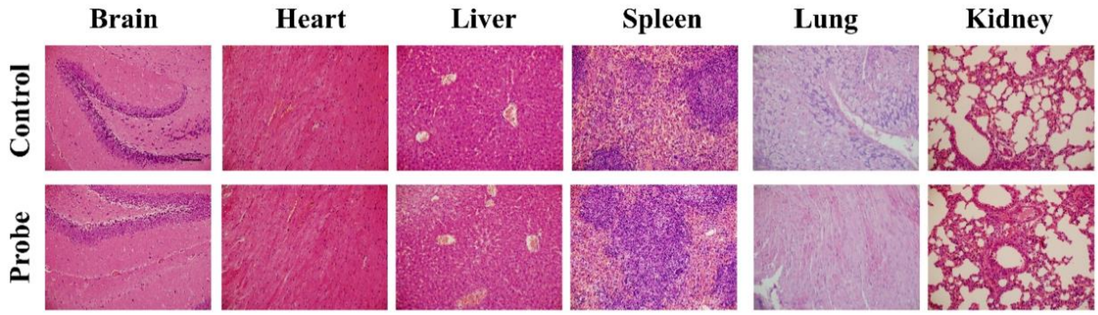

Fig. S20. H&E staining results of different organs collected from the control group and **Mito-ONOO** (200 μL, 200 μM) treated group. Scale bar: 100 μm.

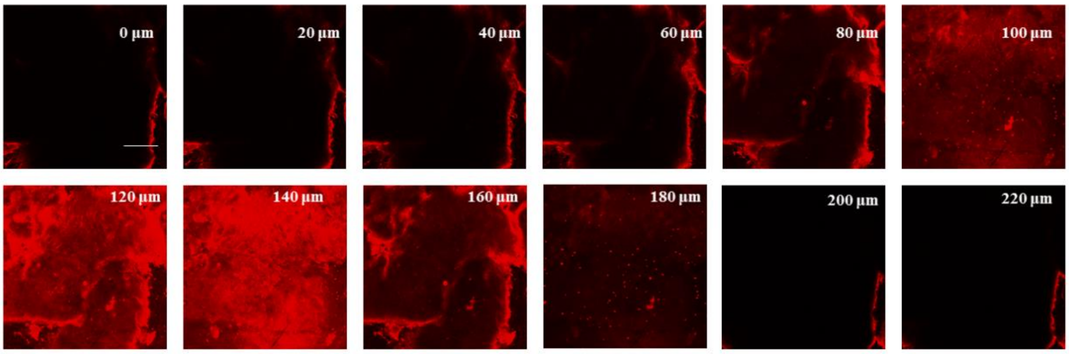

Fig. S21. Depth fluorescence images of **Mito-ONOO** (200 μL, 200 μM) in mice brain tissues. λ_{ex} = 810 nm, λ_{em} = 600–700 nm. Scale bar: 100 μm.

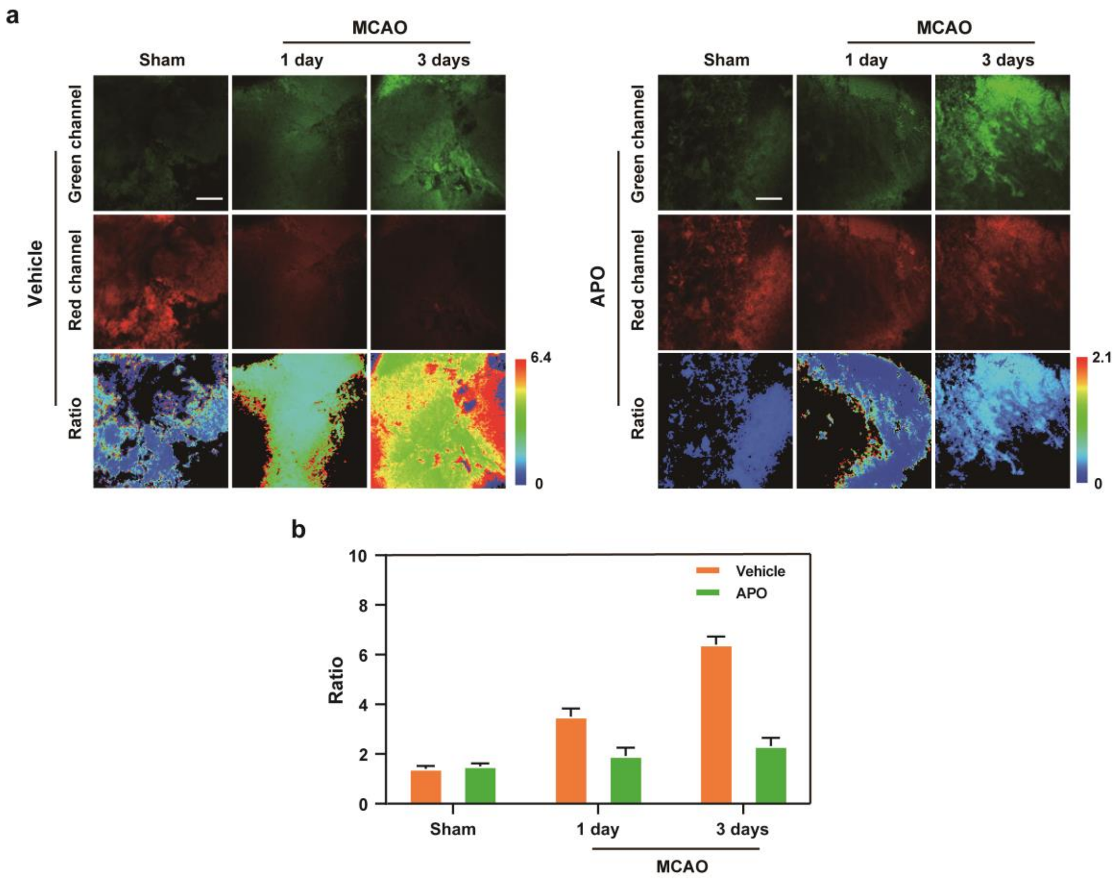

Fig. S22. a) Two-photon fluorescence imaging of stroke in brain tissues at different days (**Mito-ONOO**: 200 μL, 200 μM). b) Mean intensity ratio (F_{green}/F_{red}) of a). Emissions were collected using the green channel (450-530 nm) and red channel (600-700 nm) λ_{ex} = 810 nm. Scale bar: 200 μm. Data represent the mean of three replicates and the error bars indicate the SD.

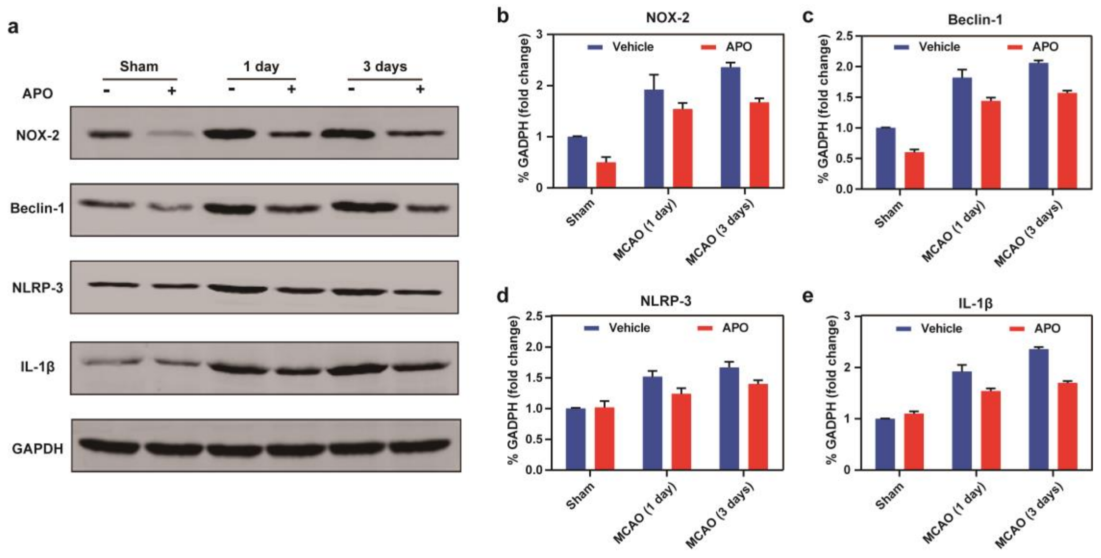

Fig. S23. a) Western blotting illustrates the expression of NOX-2, Beclin-1, NLRP-3, IL-1β and GAPDH from samples in Fig. 5a. Quantification data of the Western blot results of NOX-2 b), Beclin-1 c), NLRP-3 d) and IL-1β e) from samples in Fig. 5a. The error bars indicate the SD.

4. Supplementary Table

Table S1. The photophysical properties of **1** in various solvents.

Solvent	λ_{max}^{abs} (nm)[a]	ε ($M^{-1}cm^{-1}$)[b]	λ_{max}^{em} (nm)[c]	Φ[d]	λ_{max}^{TP-ex} (nm)[e]	δ_{max} (GM)[f]	$\Phi\delta_{max}$ (GM)[g]
CH_3CN	407	5234	481	0.34	n.d.[h]	n.d.	n.d.
EtOH	399	6834	482	0.83	n.d.	n.d.	n.d.
DMSO	402	6952	489	0.79	n.d.	n.d.	n.d.
PBS	407	6383	490	0.01	n.d.	n.d.	n.d.
DMF	410	495	485	0.68	n.d.	n.d.	n.d.
Dioxane	403	6481	475	0.30	n.d.	n.d.	n.d.
THF	397	6870	475	0.30	n.d.	n.d.	n.d.
40% EtOH	409	7550	492	0.22	810	545	120

[a] maximum absorption wavelength. [b] maximum fluorescence emission wavelength. [c] maximum two-photon excitation wavelength. [d] molar absorptivity. [e] quantum yield. [f] two-photon absorption cross section (1 GM =1 × 10^{-50} cm^4 s $photon^{-1}$). [g] two-photon action cross section. [h] n.d. = not determined.

5. References

[1] Li, R.-Q.; Mao, Z.-Q.; Rong, L.; Wu, N.; Lei, Q.; Zhu, J.-Y.; Zhuang, L.; Zhang, X.-Z.; Liu, Z.-H. A two-photon fluorescent probe for exogenous and endogenous superoxide anion imaging in vitro and *in vivo*. *Biosens. Bioelectron.* 2017, 87, 73-80.

[2] Zeng, L. Y.; Xia, T.; Hu, W.; Chen, S. Y.; Chi, S. Y.; Lei, Y. D.; Liu, Z. H. Visualizing the regulation of hydroxyl radical level by superoxide dismutase via a specific molecular probe. *Anal. Chem.* 2018, 90, 1317-1324.

[3] Jia, X. T.; Chen, Q. Q.; Yang, Y. F.; Tang, Y.; Wang, R.; Xu, Y. F.; Zhu, W. P.; Qian, X. H. FRET-based mito-specific fluorescent probe for ratiometric detection and imaging of endogenous peroxynitrite: dyad of Cy3 and Cy5. *J. Am. Chem. Soc.* 2016, 138, 10778-10781.

[4] Mao, Z. Q.; Feng, W. Q.; Li, Z.; Zeng, L. Y.; Lv, W. J.; Liu, Z. H. NIR in, far-red out: developing a two-photon fluorescent probe for tracking nitric oxide in deep tissue. *Chem. Sci.* 2016, 7, 5230-5235.

[5] Cheng, F.; Qiang, T. T.; Ren, L. F.; Liang, T. Y.; Gao, X. Y.; Wang, B. S.; Hu, W. Observation of inflammation-induced mitophagy during stroke by a mitochondria-targeting two-photon ratiometric probe. *Analyst* 2021, 146, 2632-2637.

[6] Frisch, M. J., Trucks, G. W., Schlegel, H. B., Scuseria, G. E., Robb, M. A., Cheeseman, J. R. Scalmani, G., Barone, V., Mennucci, B., Petersson , G. A., et al. Gaussian 09, Revision A.1; Gaussian, Inc.: Wallingford, CT, 2009.

[7] Ye, M. T.; Hu, W.; He, M.; Li, C. C.; Zhai, S. Y.; Liu, Z. H.; Wang, Y. Y.; Zhang, H. J.; Li, C. Y. Deep imaging for visualizing nitric oxide in lipid droplets: discovering the relationship between nitric oxide and resistance to cancer chemotherapy drugs. *Chem. Comm.* 2020, 56, 6233-6236.

[8] Zhao, Y.; Zhang, Y.; Lv, X.; Liu, Y. L.; Chen, M. L.; Wang, P.; Liu, J.; Guo, W. Through-bond energy transfer cassettes based on coumarin–Bodipy/distyryl Bodipy dyads with efficient energy efficiences and large pseudo-Stokes' shifts. *J. Mater. Chem.* 2011, 21, 13168-13171.

[9] Zhang, Y. Y.; Li, Z.; Hu, W.; Liu, Z. H. A mitochondrial-targeting near-infrared fluorescent probe for visualizing and monitoring viscosity in live cells and tissues. *Anal. Chem.* 2019, 91, 10302-10309.

[10] Xiong, X. X.; Gu, L. J.; Wang, Y.; Luo, Y.; Zhang, H. F.; Lee, J.; Krams, S.; Zhu, S. M.; Zhao, H. Glycyrrhizin protects against focal cerebral ischemia via inhibition of T cell activity and HMGB1-mediated mechanisms. *J. Neuroinflammation.* 2016, 13, 241.

[11] Xiong, X. X.; Xu, L.; Wei, L.; White, R. E.; Ouyang, Y.-B.; Giffard, R. G. IL-4 is required for sex differences in vulnerability to focal ischemia in mice. *Stroke* 2015, 46, 2271-2276.

[12]. Lee, S. K.; Yang, W. J.; Choi, J. J.; Kim, C. H.; Jeon, S. J.; Cho, B. R.; 2,6-Bis[4-(p-dihexylaminostyryl)styryl]anthracene Derivatives with Large Two-Photon Cross Sections, *Org. Lett.*, **2005**, 7, 323-326.

[13]. Li, C. C.; Hu, W.; Wang, J. C.; Song, X. J.; Xiong, X. X.; Liu, Z. H.; A highly specific probe for the imaging of inflammation-induced endogenous nitric oxide produced during the stroke process, *Analyst*, **2020**,145, 6125-6129.

[14] Gu, L. J.; Xiong, X. X.; Zhang, H. F.; Xu, B. H.; Steinberg, G. K.; Zhao, H. Distinctive effects of t cell subsets in neuronal injury induced by cocultured splenocytes *in vitro* and by *In vivo* stroke in mice. *Stroke* 2012, 43, 1941-1946.

[15] Xiong, X. X.; Xie, R.; Zhang, H. F.; Gu, L. J.; Xie, W. Y.; Cheng, M.; Jian, Z. H.; Kovacina, K.; Zhao, H. PRAS40 plays a pivotal role in protecting against stroke by linking the Akt and mTOR pathways. *Neurobiol. Dis.* 2014, 66, 43-52.

[16] Stary, C. M.; Xu, L. J.; Li, L.; Sun, X. Y.; Ouyang, Y.-B.; Xiong, X. X.; Zhao, J.; Giffard, R. G. Inhibition of miR-181a protects female mice from transient focal cerebral ischemia by targeting astrocyte estrogen receptor-α. Mol. *Cell. Neurosci.* 2017, 82, 118-125.

[17 Han, R.-Q.; Ouyang, Y.-B.; Xu, L. J.; Agrawal, R.; Patterson, A. J.; Giffard, R. G. Postischemic brain injury is attenuated in mice lacking the β2-adrenergic receptor. *Anesth. Analg.* 2009, 108, 280-287.

[18] Fang, W. R.; Zhai, X.; Han, D.; Xiong, X. X.; Wang, T.; Zeng, X.; He, S. C.; Liu, R.; Miyata, M.; Xu, B. H.; Zhao, H. CCR2-dependent monocytes/macrophages exacerbate acute brain injury but promote functional recovery after ischemic stroke in mice. *Theranostics* 2018, 8, 3530-3543.

[19]. Terao, J.; Nagao, A.; Park, D. K.; Lim, B. P.; Lipid hydroperoxide assay for antioxidant activity of carotenoids, *Methods Enzymol.*, **1992**, 213, 454-460.

[20]. Kohen, R.; Yamamoto, Y.; Cundy, K. C.; Ames, B. N.; Antioxidant activity of carnosine, homocarnosine, and anserine present in muscle and brain, *Proc. Natl. Acad. Sci. U. S. A.*, **1988**, 85, 3175-3179.

6. ^{1}H NMR Spectra, ^{13}C NMR Spectra, and HRMS Spectra of Compounds

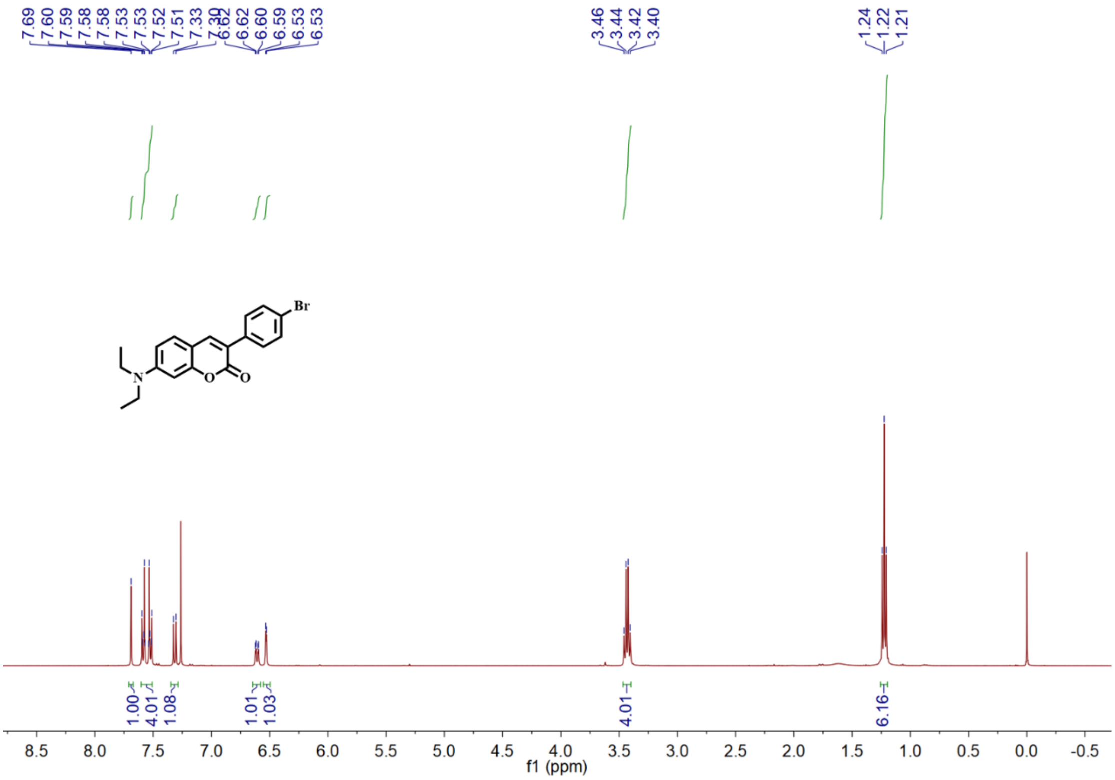

Fig. S24. The ^{1}H NMR spectrum (400 MHz) of **1** in $CDCl_3$.

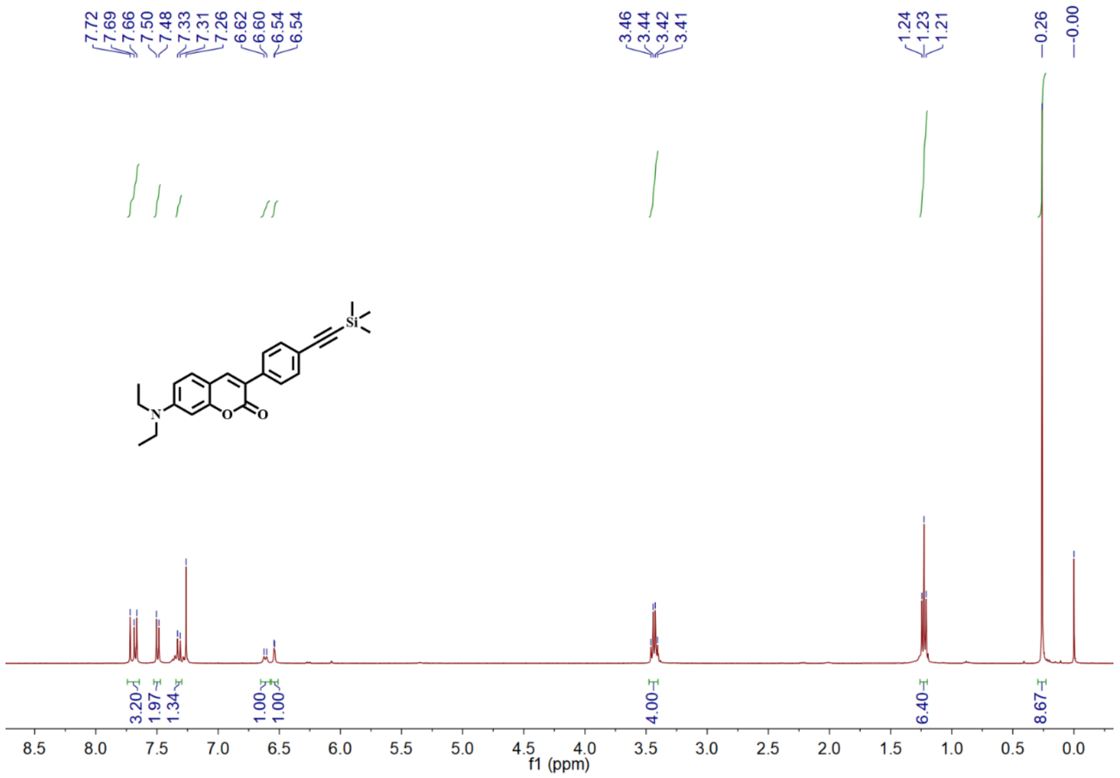

Fig. S25. The ^{1}H NMR spectrum (400 MHz) of **2** in $CDCl_3$.

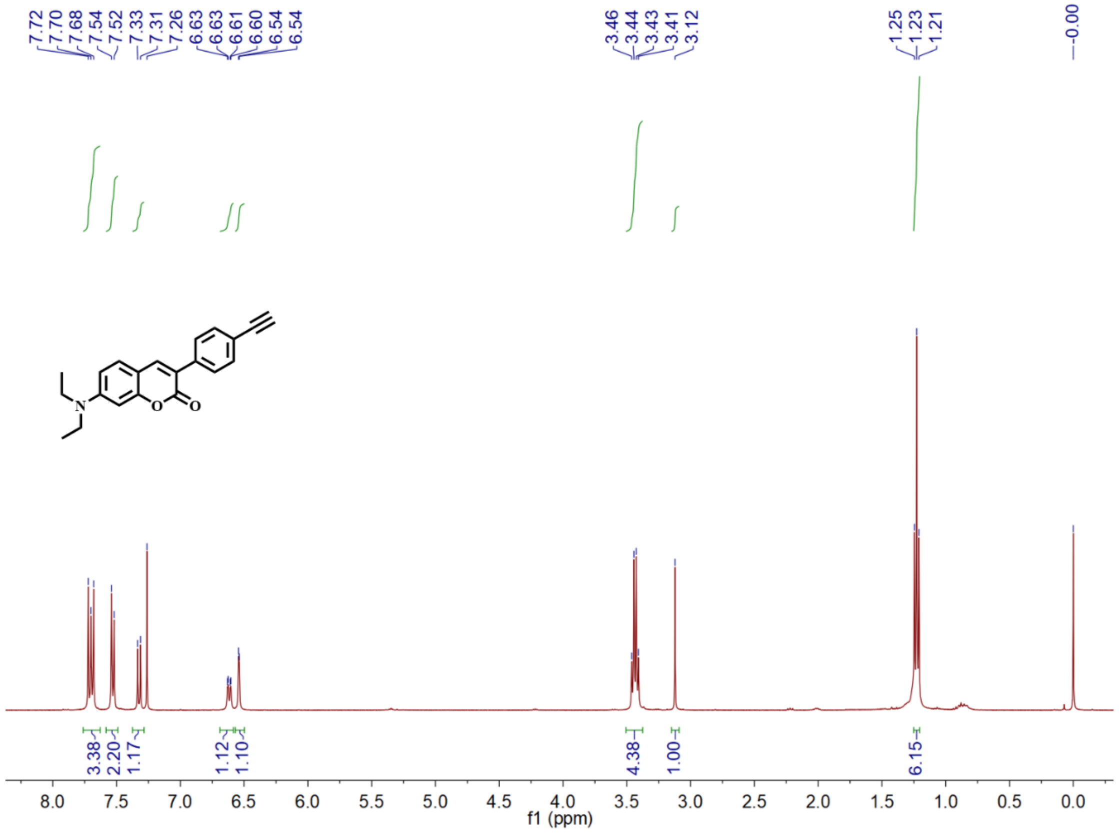

Fig. S26. The ^{1}H NMR spectrum (400 MHz) of **3** in $CDCl_3$.

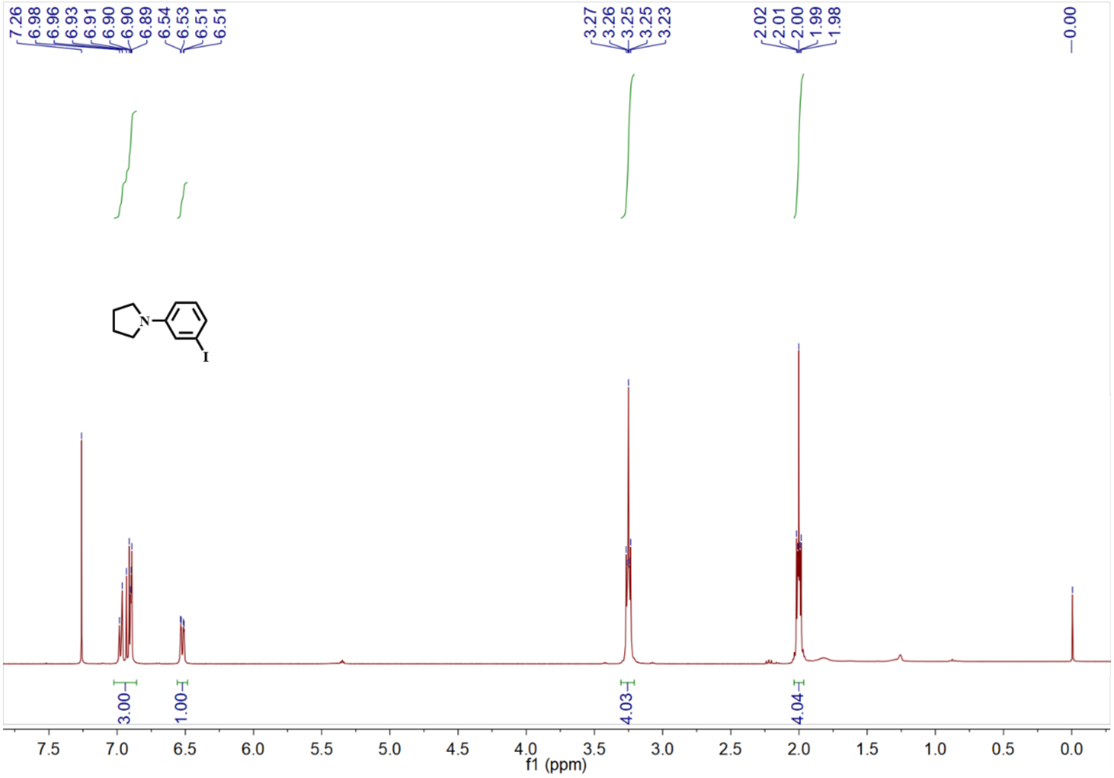

Fig. S27. The ^{1}H NMR spectrum (400 MHz) of **4** in $CDCl_3$.

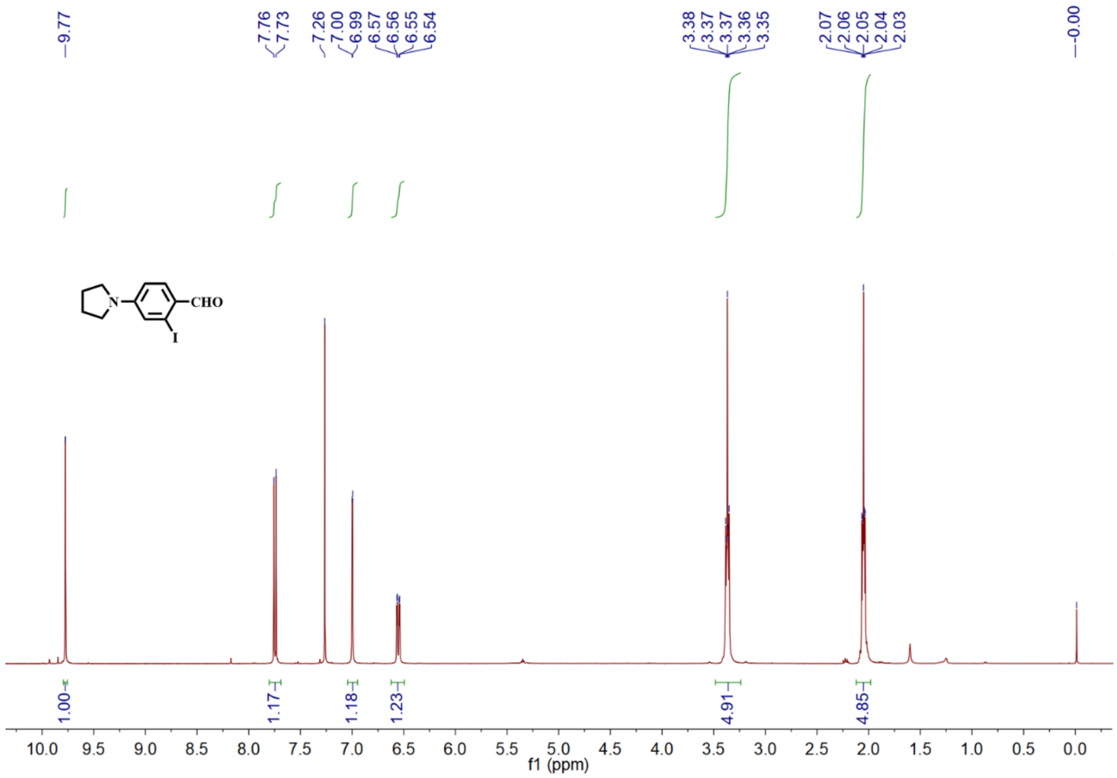

Fig. S28. The ^{1}H NMR spectrum (400 MHz) of **5** in $CDCl_3$.

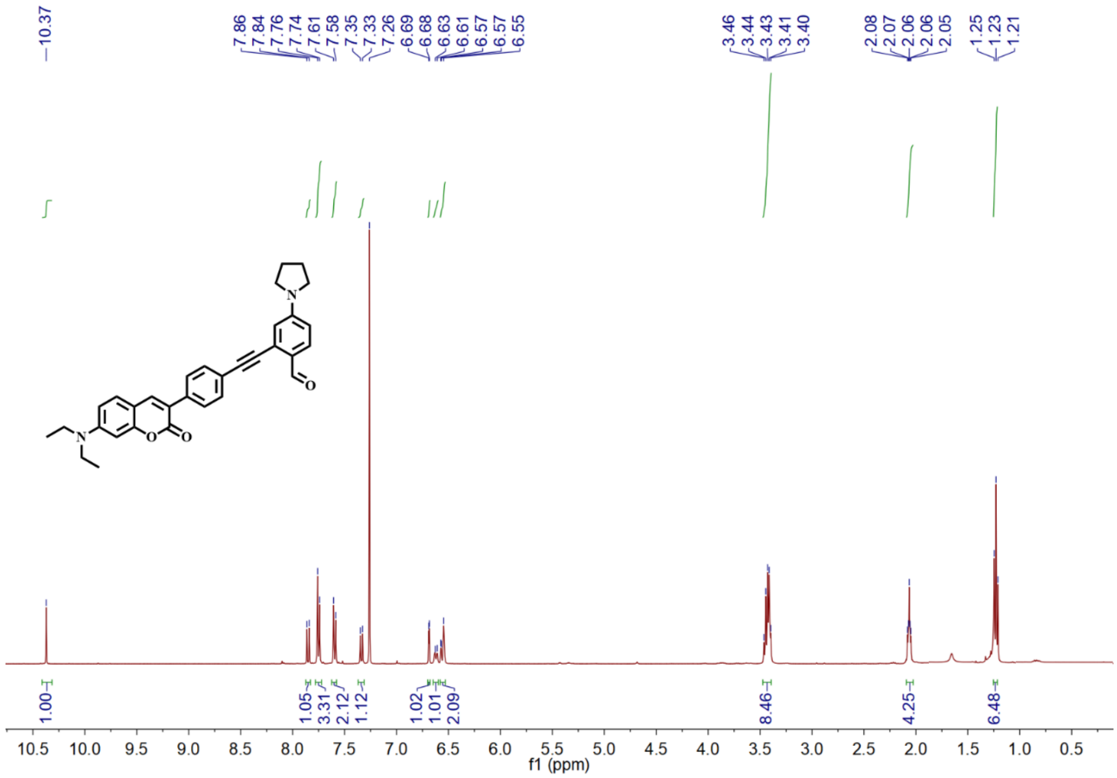

Fig. S29. The ^{1}H NMR spectrum (400 MHz) of **6** in $CDCl_3$.

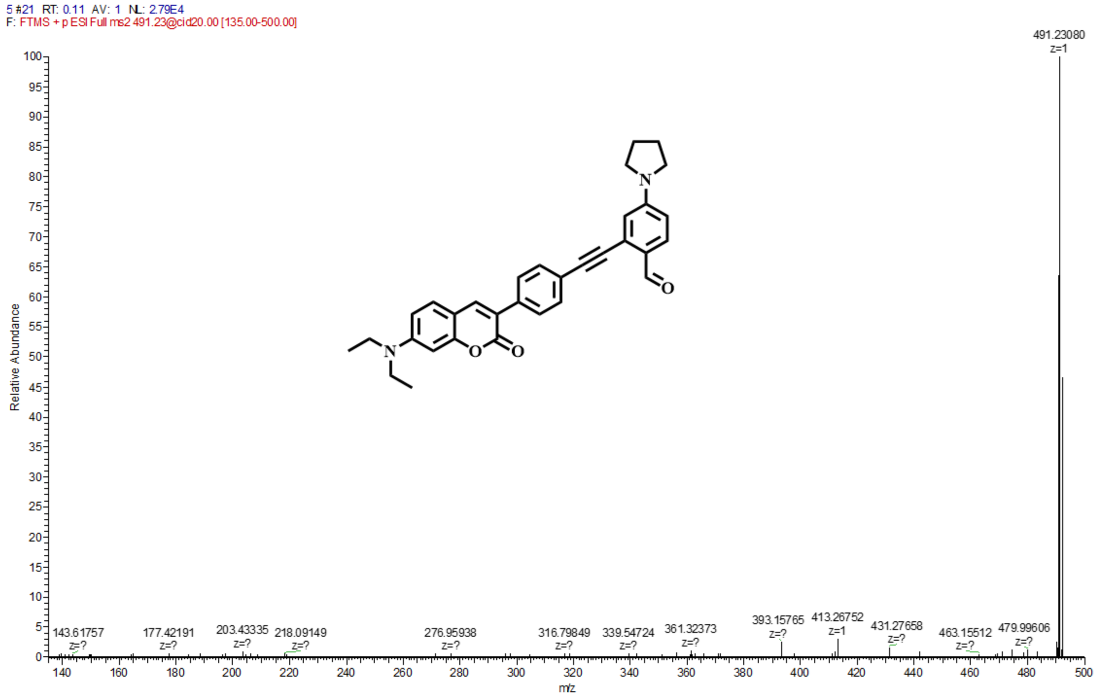

Fig. S30. The HR-MS spectrum of **6**.

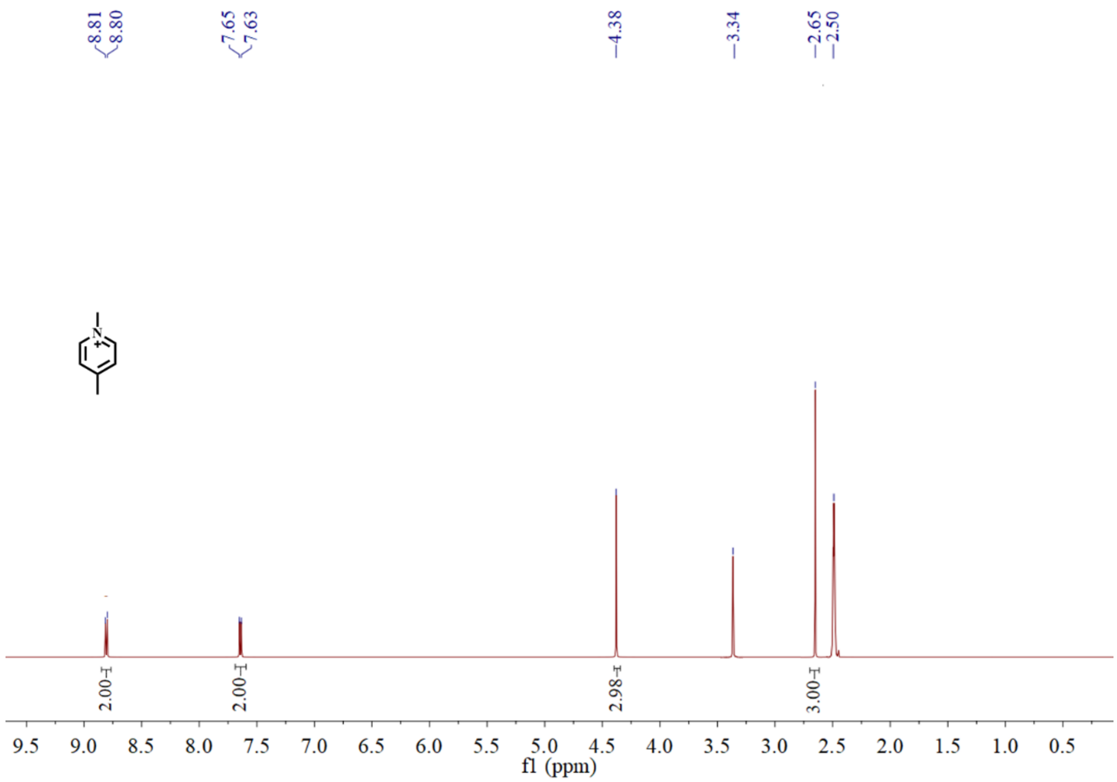

Fig. S31. The ^{1}H NMR spectrum (400 MHz) of **7** in DMSO-d_6

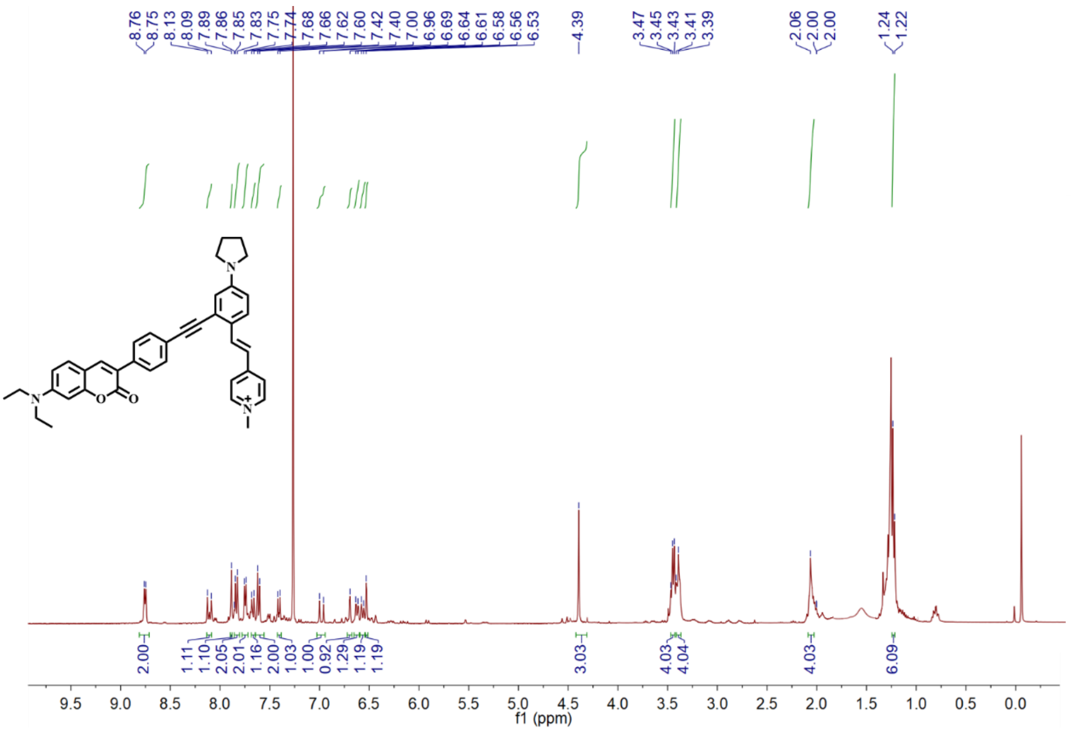

Fig. S32. The ^{1}H NMR spectrum (400 MHz) of **Mito-ONOO** in $CDCl_3$.

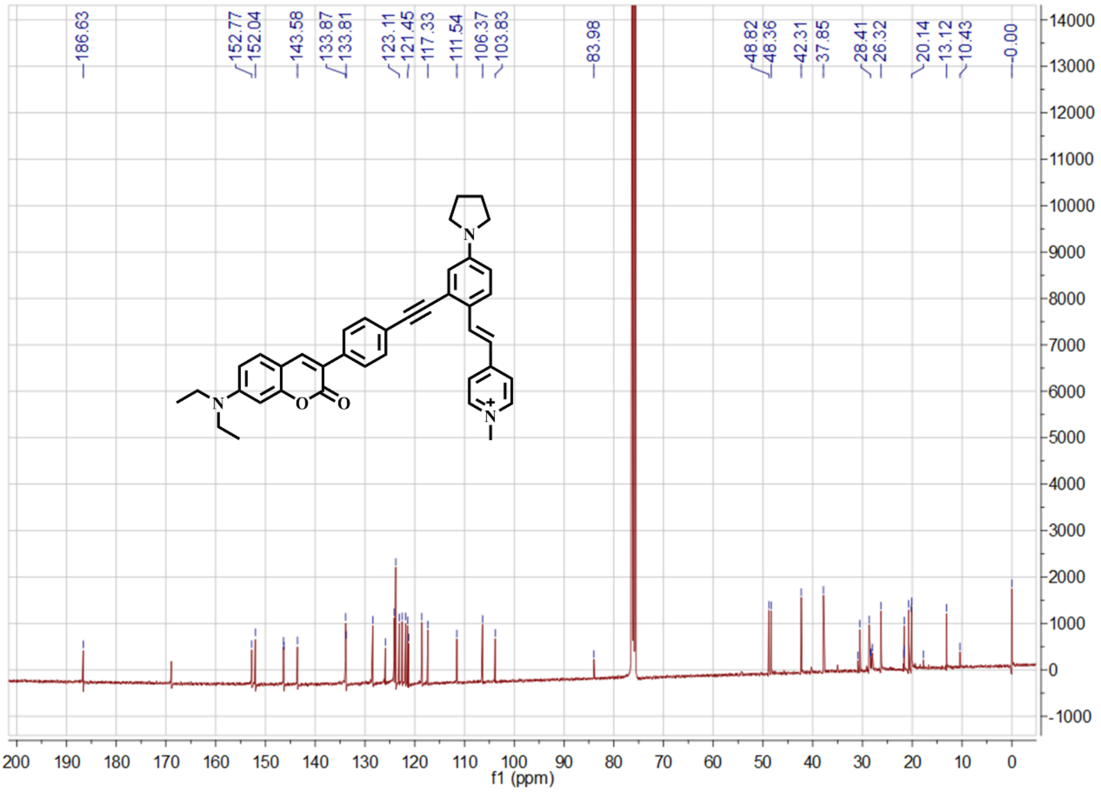

Fig. S33. The ^{13}C NMR spectrum (100 MHz) of **Mito-ONOO** in $CDCl_3$.

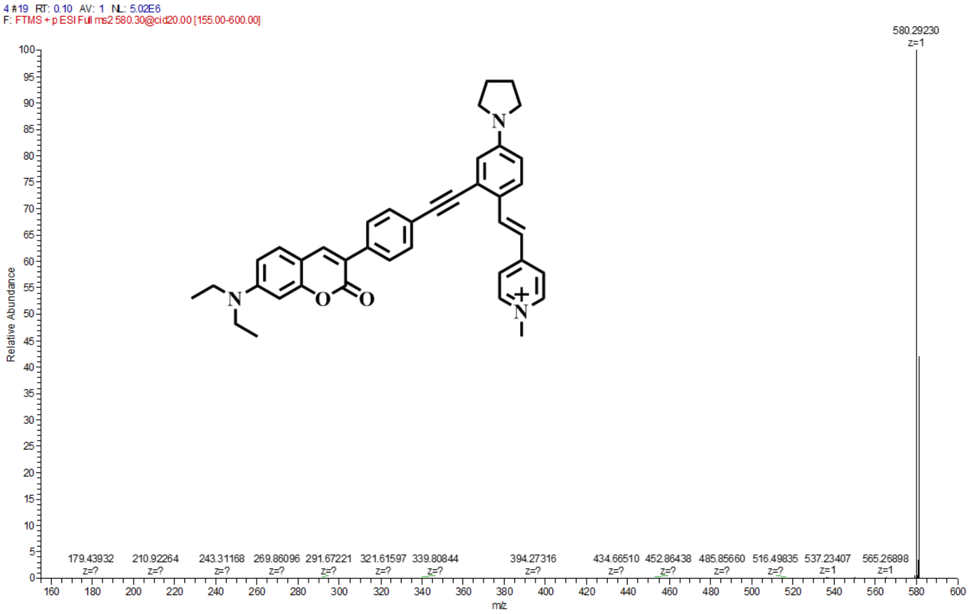

Fig. S34. The HR-MS spectrum of **Mito-ONOO**.

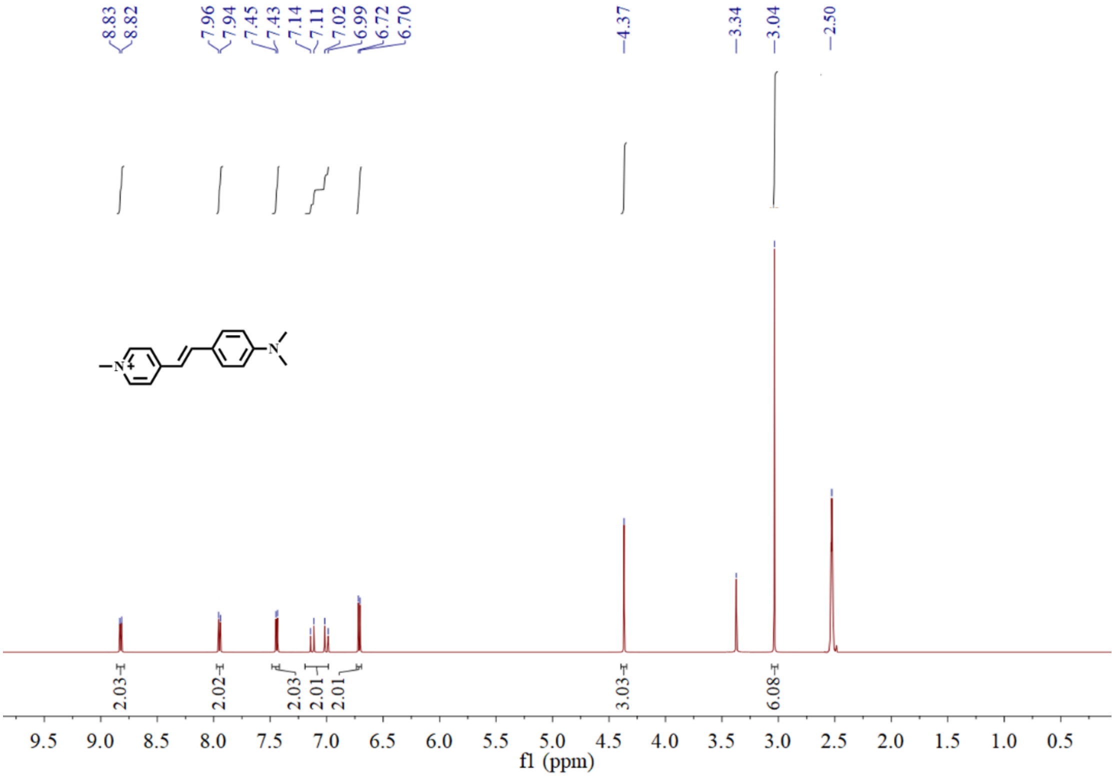

Fig. S35. The ^{1}H NMR spectrum (400 MHz) of **A-ONOO** in DMSO-d_6.
